# Crystal Structure of 4,6-α-Glucanotransferase GtfC-ΔC from Thermophilic *Geobacillus* 12AMOR1: Starch Transglycosylation in Non-Permuted GH70 Enzymes

**DOI:** 10.1021/acs.jafc.2c06394

**Published:** 2022-11-28

**Authors:** Tjaard Pijning, Evelien M. te Poele, Tijn C. de Leeuw, Albert Guskov, Lubbert Dijkhuizen

**Affiliations:** † Biomolecular X-ray Crystallography, Groningen Biomolecular Sciences and Biotechnology Institute (GBB), University of Groningen, Nijenborgh 7, 9747 AG Groningen, The Netherlands; ‡ Microbial Physiology, Groningen Biomolecular Sciences and Biotechnology Institute (GBB), University of Groningen, Nijenborgh 7, 9747 AG Groningen, The Netherlands; § CarbExplore Research B.V., Zernikelaan 8, 9747 AA Groningen, The Netherlands

**Keywords:** GtfC, α-glucanotransferase, Glycoside Hydrolase Family 70, *Geobacillus*, α-1,4/α-1,6 alternan

## Abstract

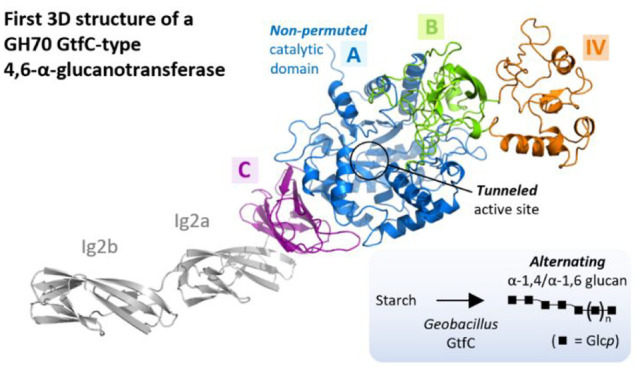

GtfC-type 4,6-α-glucanotransferase (α-GT) enzymes from Glycoside Hydrolase Family 70 (GH70) are of interest for the modification of starch into low-glycemic index food ingredients. Compared to the related GH70 GtfB-type α-GTs, found exclusively in lactic acid bacteria (LAB), GtfCs occur in non-LAB, share low sequence identity, lack circular permutation of the catalytic domain, and feature a single-segment auxiliary domain IV and auxiliary C-terminal domains. Despite these differences, the first crystal structure of a GtfC, GbGtfC-ΔC from *Geobacillus* 12AMOR1, and the first one representing a non-permuted GH70 enzyme, reveals high structural similarity in the core domains with most GtfBs, featuring a similar tunneled active site. We propose that GtfC (and related GtfD) enzymes evolved from starch-degrading α-amylases from GH13 by acquiring α-1,6 transglycosylation capabilities, before the events that resulted in circular permutation of the catalytic domain observed in other GH70 enzymes (glucansucrases, GtfB-type α-GTs). AlphaFold modeling and sequence alignments suggest that the GbGtfC structure represents the GtfC subfamily, although it has a so far unique alternating α-1,4/α-1,6 product specificity, likely determined by residues near acceptor binding subsites +1/+2.

## Introduction

Starch is a major energy-providing ingredient in many of our foods; it is digested by starch-degrading human enzymes in the gastrointestinal tract. The action of these enzymes, such as α-amylases and glucosidases, may result in an undesirably rapid release of glucose in the blood, increasing the risk of cardiovascular diseases in the long term.^1^ To lower such risks, the food industry is aiming to produce starch-based products with altered molecular structure, endowing prebiotic properties.^1−6^ The 4,6-α-glucanotransferase (4,6-α-GT) enzymes from Glycoside Hydrolase Family 70 (GH70) provide a promising strategy to modify starch in this way as they introduce α-1,6 glycosidic linkages, resulting in a slower degradation.^7−10^ The first characterized GH70 4,6-α-GTs were found in lactic acid bacteria (LAB)^11−17^ and designated the GH70 GtfB subfamily. More recently, however, enzymes with 4,6-α-GT reaction specificity were also characterized in non-LAB species, sharing low sequence similarity with GtfB enzymes (<30%); these were designated the GtfC subfamily.^18^ Of the 30 putative GtfC enzymes found in public databases by 2018,^2^ four have been biochemically characterized;^18−22^ among them are enzymes from thermophilic bacteria, increasing the potential of these enzymes in an industrial setting, as they were able to convert starch into linear isomalto-/maltooligosaccharides at high temperatures (60–68 °C).^22^ For example, adding the *Geobacillus* 12AMOR1 GtfC (GbGtfC) enzyme during bread baking showed antistaling effects. In addition to GtfCs, a few 4,6-α-GT enzymes with even lower sequence similarity were identified in (plant-associated) bacteria. The characterized enzymes in this group synthesized reuteran-like branched α-glucans instead of linear products,^23,24^ thus defining another GH70 4,6-α-GT subfamily (GtfD).

The transglycosylation reaction catalyzed by GH70 4,6-α-GTs involves three catalytic residues (two Asp and one Glu) and has been described by two half-reactions, each involving an oxocarbenium-ion type transition state, stabilized by an Asp residue. The first half-reaction is α-1,4 specific cleavage of the substrate and results in a covalent enzyme-glycosyl intermediate, which is transferred with α-1,6 specificity to an acceptor substrate in the second half-reaction, leading to isomalto-/maltooligosaccharide (IMMO/IMMP) products containing α-1,6 linked units at the non-reducing end. Previously, we structurally characterized 4,6-α-GT enzymes of the GtfB subfamily^25,26^ and proposed a reaction scheme involving sliding of intermediate products through the binding groove. We then hypothesized that the substrate and product specificity of different GtfB-type 4,6-α-GTs is related to the accessibility of the active site binding groove, which is defined by two loops (A1 and B). A (phylogenetic) survey suggested that about 80% of enzymes in the GtfB subfamily feature a tunneled binding groove,^26^ while the remaining ones are (much) more open, allowing for the processing of branched substrates and products. The so far characterized GtfC-type 4,6-α-GTs generated linear products, although it has to be noted that the tested substrates were also largely linear. To date, no GtfC protein 3D structures have been reported; given the low sequence identity with GtfB-type 4,6-α-GTs (<30%) the question is whether GtfCs feature a tunnel or not and if a similar diversity with regard to active site openness exists. Interestingly, the GtfC from *Geobacillus* 12AMOR1 (GbGtfC) was found to have a unique product specificity.^22^ With a limited hydrolytic activity, GbGtfC releases mainly maltose instead of glucose from amylose V or maltoheptaose substrate, synthesizing a main product containing alternating α-1,4/α-1,6 linkages instead of consecutive α-1,6 linkages. This suggests that GbGtfC exclusively transfers maltosyl units instead of glucosyl units, but the structural details that confer this property remain to be uncovered.

Importantly, the GtfC-type 4,6-α-GTs differ from their GtfB-type relatives (and GH70 glucansucrases) regarding domain organization. First, GtfCs lack the circular permutation of the (β/α)_8_-barrel in the catalytic domain A, as is the case in GH13 α-amylases belonging to the same clan GH-H.^18,27,28^ Despite this absence of permutation, all seven conserved sequence regions I–VII found in GH-H enzymes were predicted to be present.^18^ Second, GtfCs were predicted to lack domain V and to have a single-segment domain IV. This domain IV was proposed to have been inserted into domain B of an ancestor α-amylase of the GH13_5 subfamily, which mainly originate from bacteria and also act on starch-like substrates, but lack this domain IV.^2,18,25,28^ Finally, some GtfC-type enzymes were predicted to feature additional C-terminal domains of the bacterial Ig (type 2) fold.^2,18^ Phylogenetic analysis and predicted domain organization lead to the hypothesis that the GtfC subfamily represents an intermediate in a linear evolutionary pathway between GH13_5 α-amylases and GtfB-type 4,6-α-GTs.^2,18^ Yet, since no GtfC 3D structures have been reported, it is still unknown whether GtfCs resemble more the α-amylases or the GtfBs structurally.

Here, we report the first crystal structure of a GtfC-type enzyme, the 4,6-α-GT from *Geobacillus* 12AMOR1 (GbGtfC), revealing the 3D structure of the core domains A, B, C, and the single-segment domain IV. Despite the absence of circular permutation, GbGtfC features a tunneled active site architecture that closely resembles the majority of GtfB-type 4,6-α-GTs. The obtained structure of the GbGtfC-ΔC enzyme (at 2.25 Å resolution), together with docking experiments depicting donor and acceptor reactions, allowed us to pinpoint the residues in the active site that likely contribute to its unique “alternating” specificity. AlphaFold modeling confirmed that GbGtfC features two C-terminal domains of the Ig (type 2) fold that are absent in the crystallized construct. Finally, we show that the GbGtfC 3D structure represents the GtfC α-GT subfamily as currently known, suggesting that the structural changes necessary to acquire the α-1,6 starch-transglycosylating specificity of GH70 α-GTs from starch-degrading GH13 α-amylases took place before domain permutation events.

## Materials and Methods

### Expression and Purification

The cloning and expression of the GbGtfC-ΔC construct, containing residues 33–738 of *Geobacillus* 12AMOR1 GtfC and a 20-residue N-terminal His-tag, have been described before.^22^ Briefly, the pET15b vector carrying the *gtf*C construct was overexpressed in *E. coli* BL21 (DE3) cultures grown at 37 °C; harvested cells were resuspended and broken by sonication; cell-free extract (CFE) was stored at 4 °C. The GbGtfC-ΔC protein in the CFE was captured by immobilized metal affinity chromatography (IMAC) on a Ni-Sepharose column (Sigma-Aldrich, St. Louis, MO) using an elution buffer containing 20 mM Tris-HCl, pH 8.0, 100 mM NaCl, and 350 mM imidazole. Fractions with the highest absorbance at 280 nm were pooled and concentrated using a VivaSpin 4 (molecular weight cutoff 10 kDa) at 4000*g*. The final purification step was done *via* size exclusion chromatography on an Äkta Micro system equipped with a Superdex 200 Increase 10/300 column (Cytiva, Marlborough, MA) at 12 °C. The elution buffer contained 20 mM MES-NaOH, pH 6.1, 100 mM NaCl, and 1 mM CaCl_2_. The center fractions of the peak eluting at 13.3–14.8 mL were pooled (Figure S1) and concentrated as described above to obtain the final GbGtfC-ΔC protein sample suitable for crystallization. Protein concentrations were determined by measuring the absorbance at 280 nm using a NanoDrop One spectrophotometer (Isogen Life Science, De Meern, The Netherlands).

### Crystallization and Data Collection

Crystals of GbGtfC-ΔC were grown at 20 °C using a 10.0 mg/mL protein solution, 20 mM MES-NaOH, pH 6.1, 100 mM NaCl, and 1 mM CaCl_2_. The reservoir solution contained 1.07–1.14 M (NH_4_)_2_SO_4_, 0.1 M MES-NaOH, pH 6.5, and 0.4 M Na_3_citrate, and hanging drops were prepared by mixing 1.5 μL of protein solution and 1.5 μL of reservoir solution. Prior to data collection, crystals were briefly transferred to 1.25 M (NH_4_)_2_SO_4_, 0.05 M MES-NaOH, pH 6.5, 0.2 M Na_3_citrate, and 30% (v/v) glycerol and flash-cooled in liquid nitrogen. X-ray diffraction data were collected at beamline I03 of the Diamond Light Source (UK) and processed using XDS;^29^ statistics are given in Table 1.

**Table 1:** Crystallographic Data Collection and Refinement Statistics

PDB entry	7ZC0
resolution (Å)	131.3–2.25 (2.31–2.25)
space group	*I*4_1_22
cell dimensions *a, b, c* (Å)	262.6, 262.6, 72.2
unique observations^a^	59408 (4359)
redundancy^a^	1.9 (1.9)
completeness (%)^a^	99.3 (95.0)
mean *I*/σ(*I*)^a^	20.5 (4.2)
Wilson B-factor (Å^2^)	33.7
*R*_pim_^a^	0.030 (0.245)
CC_1/2_^a^	0.999 (0.795)
*R*/*R*_free_	0.252/0.292
number of non-hydrogen atoms	
protein	5667
glycerol	24 (4 × 6)
Ca^2+^/water	1/257
average B-factors	
protein (Å^2^)	55.0
glycerol (Å^2^)	43.5
Ca^2+^/water (Å^2^)	21.3/39.0
root mean square deviations	
bond lengths (Å)	0.006
bond angles (deg)	1.38
Ramachandran	
favored (%)	92.0
allowed (%)	7.0
outliers (%)	1.0

a Values in parentheses represent the highest resolution shell.

### Structure Determination and Refinement

The crystal structure of GbGtfC-ΔC was determined by the molecular replacement method using PHASER;^30^ a template model was generated by the one-to-one protocol of Phyre^31^ based on the highest scoring structure from a Phyre search, the crystal structure of the α-amylase from *Halothermothrix orenii* (PDB: 3BC9).^32^ The asymmetric unit of the *I*4_1_22 cell contains one protein molecule. Refinement and model building was carried out using Refmac^33^ and COOT;^34^ groups for TLS refinement were determined using Phenix^35^ and were edited manually to include domain IV as a separate TLS group. The B-factor distribution showed a large range of values, with relatively high values for domains C and IV (Figure S2). Some stretches of residues in domain IV lacked good electron density, especially residues 271–282, which were later modeled guided by an AlphaFold generated model.^36,37^

The final refinement statistics and model quality are listed in Table 1. Structural figures were prepared with PyMOL (The PyMOL Molecular Graphics System, Version 2.0 Schrödinger, LLC). DSSP^38^ was used to define secondary structure. Atomic coordinates and structure factors have been deposited at the Protein Data Bank with entry 7ZC0. PDBeFold^39^ was used to analyze structural similarities, with a lowest acceptable match threshold of 70% or 40%.

### AlphaFold Modeling of GbGtfC and Homologues

The full sequence of GbGtfC (GenBank AKM18207.1, 903 amino acid residues) was subjected to AlphaFold modeling.^36,37^ The model with the highest overall pLDDT (per-residue confidence) score was used for comparison with the crystal structure.

Additionally, AlphaFold models were calculated for 4 other GtfC-type enzymes (Table S1), from *Heyndrickxia sporothermodurans* (902 residues), *Weizmannia coagulans* DSM1 (954 residues), *Exiguobacterium sibiricum* 255-15 (893 residues), and *Exiguobacterium acetylicum* (892 residues).

### Modeling Donor Substrate Binding

We used the native crystal structure to map the substrate binding groove of GbGtfC-ΔC; an initial model was obtained by superposition with maltoheptaose (G7) bound to subsites +2 to −5 of Lr121 GtfB^25^ and inspected in PyMOL. We then adjusted the glycosidic torsion angles of glucosyl units in further subsites, to fit the binding groove of GbGtfC-ΔC without clashes. An extra glucosyl moiety was added at the reducing end (subsite +3), yielding a final maltooctaose (G8) model. The corresponding residues from four other GtfC enzymes (*H. sporothermodurans*, *E. sibiricum* 255-15, *E. acetylicum*, and *W. coagulans* DSM1), as well as a GtfB-type 4,6-α-GT from *L. reuteri* 121 (Q5SBM0), were selected for a sequence alignment with ESPript 3.0.^40^

### Molecular Docking

Mixed isomalto-maltooligosaccharides (DP1–6) were setup using SWEET2^41^ and AutoDock Tools (version 1.5.6)^42^ and docked in the crystal structures of GbGtfC (this study) and Lr121 GtfB^25^ using Vina-Carb,^43^ representing scenarios for the donor reaction or for the acceptor reaction with a covalent glucosyl-enzyme intermediate at the catalytic nucleophile D413. All docking results were visually inspected in PyMOL, judged by hydrogen-bond interactions with catalytic residues, and then grouped by visual similarity. Details of the docking procedures and interpretation of the results are given in the Supporting Information.

### Phylogenetic Analysis

A BLASTp search with default parameters was performed (January 18, 2022) with the sequence of *Geobacillus* 12AMOR1 GtfC (Genbank AKM18207.1). Using the full sequences of the resulting hits, multiple sequence alignments were performed with MUSCLE^44^ and inspected within JalView 2;^45^ sequences lacking significant parts of the GH70 core (containing the conserved sequence regions (motifs) I–VII) were deleted. This initial alignment was extended by three extra sets of sequences representing biochemically characterized bacterial enzymes: (a) eight canonical α-amylases from GH13 subfamily 5 (GH13_5); (b) five GH70 glucansucrase sequences; and (c) six GH70 GtfB sequences.^26^ The sequences used for the final alignment are shown in Table S2. Residues constituting three important loops in GH70 GtfB-type 4,6-α-GTs were identified on the basis of previously determined structures:^25,26^ loop B in domain B and loops A1 and A2 in domain A (note that, in non-permuted GH70 sequences, loop A1 is C-terminal to loop A2). A phylogenetic tree was constructed in MEGA X^46^ using the Maximum Likelihood method; the tree with the highest log likelihood was used. Initial tree(s) for the heuristic search were obtained automatically by applying Neighbor-Join and BioNJ algorithms to a matrix of pairwise distances estimated using a JTT model and then selecting the topology with superior log likelihood value. Branch lengths were measured in the number of substitutions per site. All positions with less than 95% site coverage were eliminated, i.e., fewer than 5% alignment gaps, missing data, and ambiguous bases were allowed at any position (partial deletion option). There was a total of 512 positions in the final data set. The bootstrap consensus tree was inferred from 1000 bootstrap replicates.^47^

For a comparison between acceptor subsite residues in GtfC- and GtfB-type 4,6-α-GTs, the 63 putative GtfC sequences were aligned with a subset of the 283 putative GtfB sequences from Pijning et al.;^26^ this subset contained 233 sequences with long loops A1 and B (totaling 37–40 residues), likely featuring a tunneled binding groove.

## Results and Discussion

### Crystal Structure of GbGtfC-ΔC

#### Overall Structure

**Figure 1:**
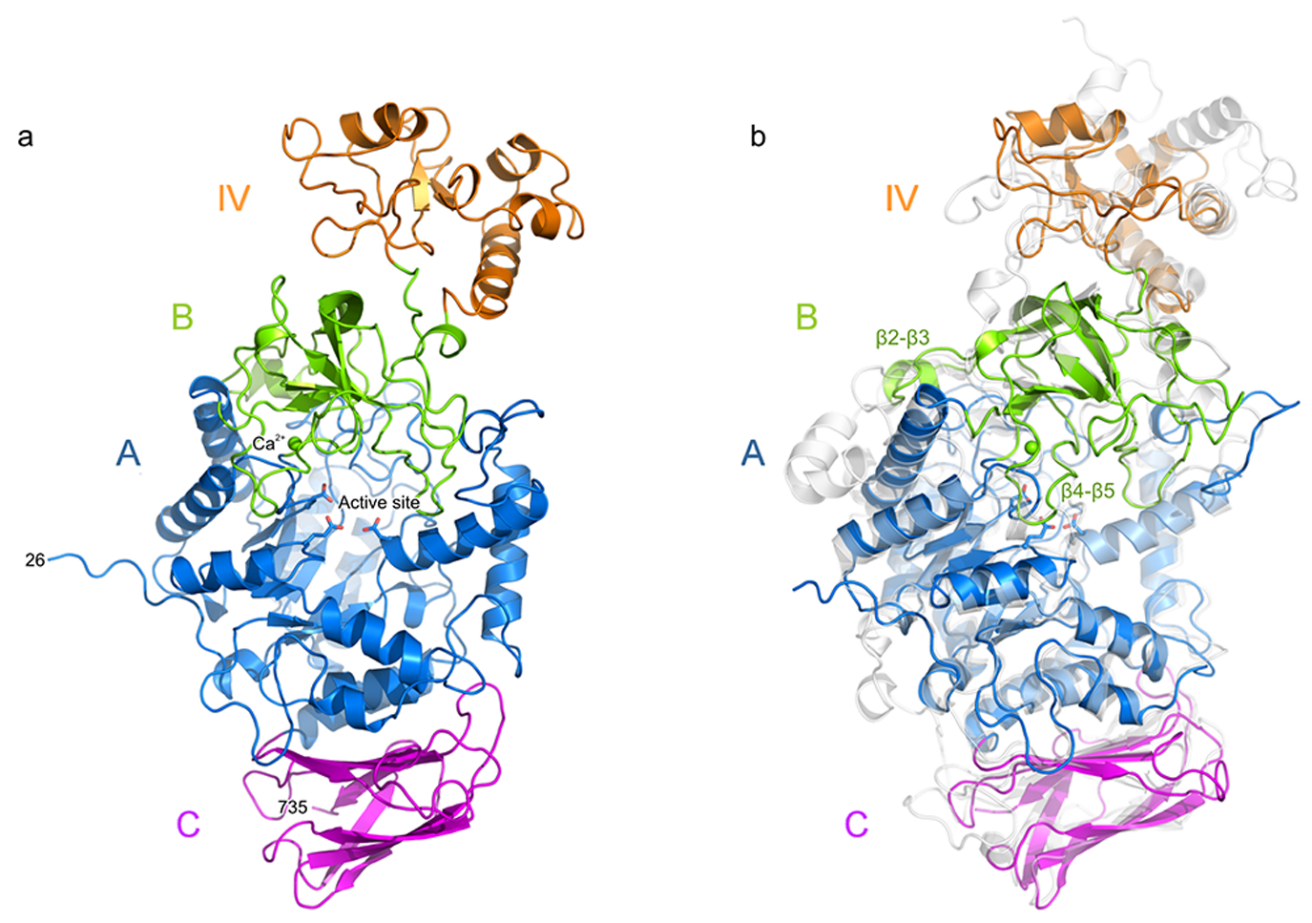
(a) Overall crystal structure of GbGtfC-ΔC, with the domains indicated. The active site is located at the interface of domains A and B, with catalytic residues D413, D446, and E517 shown as sticks. The Ca^2+^ ion near the active site is shown as a green sphere, and the first (V26) and last (K735) visible residues are indicated. (b) Superposition of the crystal structure of GbGtfC-ΔC with that of Lr121 GtfB (transparent gray; PDB: 5JBD).^25^ The Lr121 GtfB enzyme features a somewhat larger domain IV as well as longer loops in domains A, B, and C (e.g., the β2−β3 and β4−β5 connections in domain B) but shares the same overall topology.

We determined the crystal structure of GbGtfC-ΔC at a resolution of 2.25 Å from crystals containing one protomer in the asymmetric unit (Figure 1a) consisting of residues V26-K735. The crystal structure comprises domains A, B, C, and IV and is the first one representing a non-permuted GH70 enzyme. The catalytic domain A (residues 26–144 and 387–630) contains the (β/α)_8_ barrel also found in other GH70 enzymes, but, like in GH13 α-amylases, it starts with strand β1 and is interrupted after helix α3 by a long insertion, forming domain B, as well as the auxiliary domain IV, which is absent in GH13 enzymes. Despite being non-permuted, the overall topology of domain A is very similar to that of other GH70 structures (e.g., Lr121 GtfB; Figure 1b). On the other hand, some differences were observed in the elements that connect the α-helices and β-strands of the (β/α)_8_ barrel (e.g., in the β2-α2, α3-β4, and α4-β5 connection). Domain B (residues 145–222 and 333–386) has the central twisted five-stranded antiparallel β-sheet also observed in other GH70 structures but is more compact, mainly due to shorter connections between the β-strands. For example, the connection between strands β2 and β3 (residues 191–210) is about 30 residues shorter than it is in Lr121 GtfB and lacks two α-helices, while the loop connecting strands β4 and β5 (residues 357–380) is about nine residues shorter. The connection between strands β3 and β4 is “extended” by the insertion of about 110 residues that constitute domain IV (residues 223–332). Finally, domain C (residues 631–736) displays a similar Greek key topology as in other GH70 and GH13 structures, albeit some loops that connect the β-strands are either shorter or longer.

Despite the low sequence similarity, the GbGtfC-ΔC core structure closely resembles that of GtfB-type 4,6-α-GTs.^18,25,26^ Yet, PDBeFold analysis of the core domains (A, B, and C) of the GbGtfC-ΔC crystal structure revealed that the closest structural homologues are α-amylases from *Alicyclobacillus* sp. (PDB: 6GXV)^48^ and *Geobacillus stearothermophilus* (PDB: 4UZU)^49^ with Q-scores of 0.46/0.44 and root-mean-square deviations (RMSD) of 1.95/1.88 Å, respectively. Both these α-amylases belong to subfamily GH13_5, confirming structurally the previous observation that this is the α-amylase subfamily to which GH70 enzymes are evolutionary closest.^25^ Only after including domain IV to the PDBeFold search, structural homologues of GH70 enzymes were detected, the closest one being the 4,6-α-GT GtfB-ΔNΔV from *L. reuteri* 121 (Lr121 GtfB; PDB: 5JBD)^25^ with a lower Q-score (0.24) than the α-amylases but also a somewhat lower RMSD value (1.72 Å).

The GbGtfC-ΔC 3D structure confirms the earlier notion that at the domain level it represents an intermediate between GH13 α-amylases and GH70 GtfB-type α-GTs; regarding the structural details of the core domains, and especially the active site region, it is clearly similar to the GH70 GtfB-type α-GTs and more distant from the GH13 α-amylases.

#### Domain IV Structure

**Figure 2:**
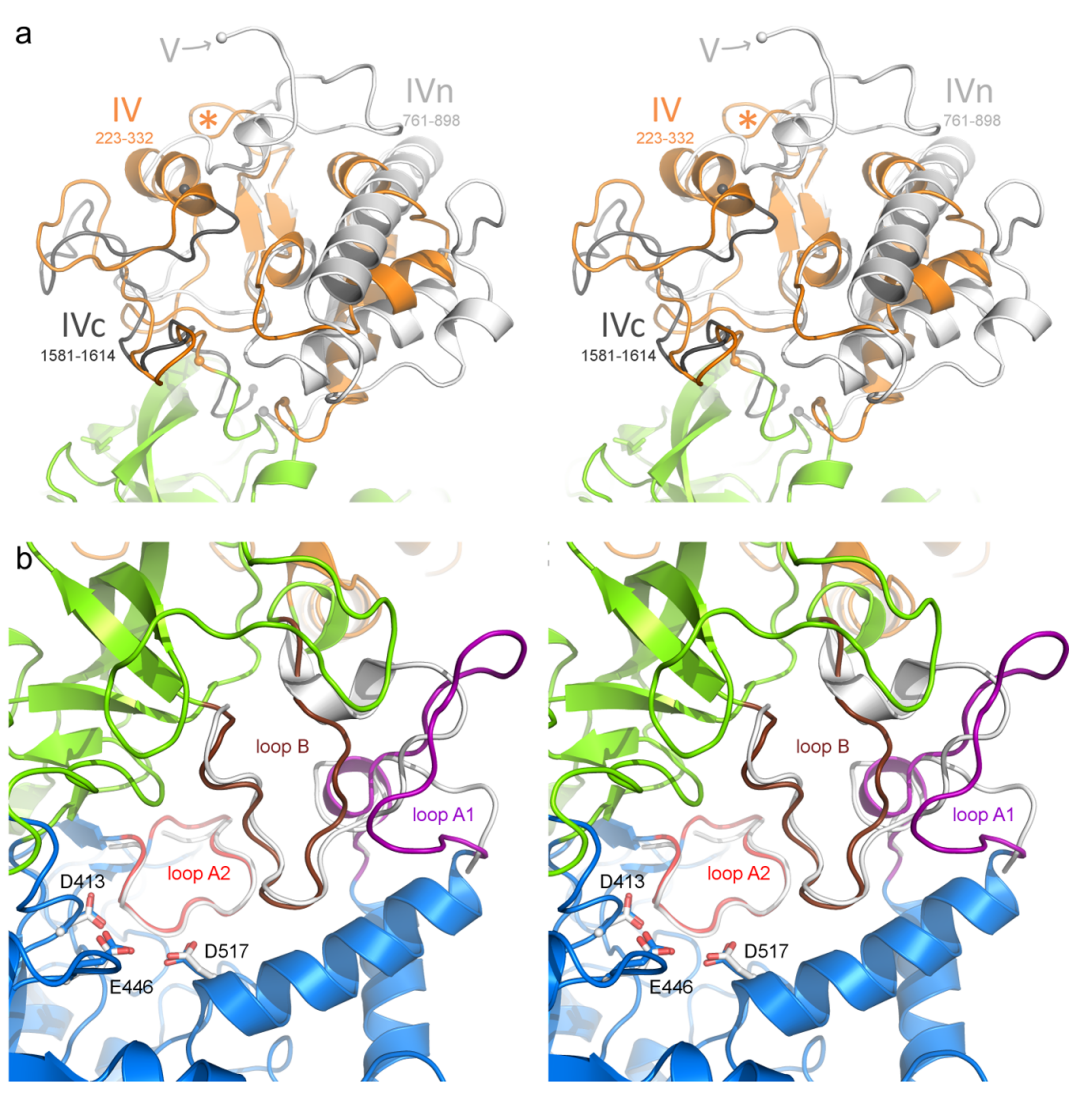
Detailed comparison of the GbGtfC-ΔC crystal structure (colored as in Figure 1; this study) with that of Lr121 GtfB-ΔNΔV (transparent gray; PDB: 5JBD).^25^ (a) Stereo figure of the superposition based on domain IV alone. In GbGtfC, the 110-residue domain IV (orange) is a single-segment insertion in domain B (green). In Lr121 GtfB, domain IV consists of an N-terminal segment (IVn, light gray) preceded by its domain V, and a C-terminal segment (IVc, dark gray) far apart in sequence, each superimposing partly with domain IV of GbGtfC-ΔC. The loop (residues 271–282 of GbGtfC-ΔC) connecting the small β-sheet is indicated with an asterisk. (b) Stereo figure of the loop architecture around the active sites; loop A1 (purple), and loop B (brown) cover donor subsites of the groove, while loop A2 (red) forms the base of the groove. The corresponding loops in Lr121 GtfB (gray) largely follow the same course. The catalytic residues are shown as sticks (in GbGtfC these are the nucleophile D413, general acid/base E446, transition state stabilizer D517).

The GbGtfC-ΔC crystal structure reveals for the first time an inserted, uninterrupted domain IV of a GH70 enzyme (Figure 2a). Domain IV comprises 110 residues (223–332) and is much smaller than the corresponding domains in GtfB-type enzymes (usually about 170–180 residues); it connects to domain B *via* two loops that lie adjacent to each other. The crystallographic B-factors for domain IV are on average higher than for other domains (Figure S2), indicating that this domain may be flexible due to a hinged connection with domain B. Some of its residues hardly showed electron density (Figure S3), but since domain IV superimposed almost perfectly with that of the AlphaFold model (see Figure 2), we confidently decided to include all residues of this domain in the crystal structure.

Notably, PDBeFold analysis of domain IV alone did not reveal significant structural similarity to known 3D structures (all Q-scores below 0.12); this domain thus can be considered a previously unobserved fold. Still, a manual inspection showed that parts of domain IV can be superimposed with that of other GH70 structures (e.g., Lr121 GtfB) (Figure 2a), taking into account that the N- and C-terminal halves of which they are composed, are “switched” due to the permutation. Indeed, the N-terminal part of domain IV of GbGtfC (residues 223–245) superimposes reasonably well with the C-terminal part of domain IV of Lr121 GtfB (residues 1586–1614), even though both lack secondary structure elements. For the other segment (GbGtfC residues 246–332), the superposition is more difficult, as the corresponding Lr121 GtfB segment (residues 761–898) features longer α-helices and longer loops. In GbGtfC domain IV, residues 271–282 form a loop at the “top” of domain IV connecting a short parallel β-sheet; a similar architecture is seen in the crystal structures of Lr121 GtfB (PDB: 5JBD)^25^ and *Limosilactobacillus reuteri* NCC2613 (Lr2613) GtfB (PDB: 7P38(26)) (albeit with longer connections).

#### Active Site and Binding Groove

**Figure 3:**
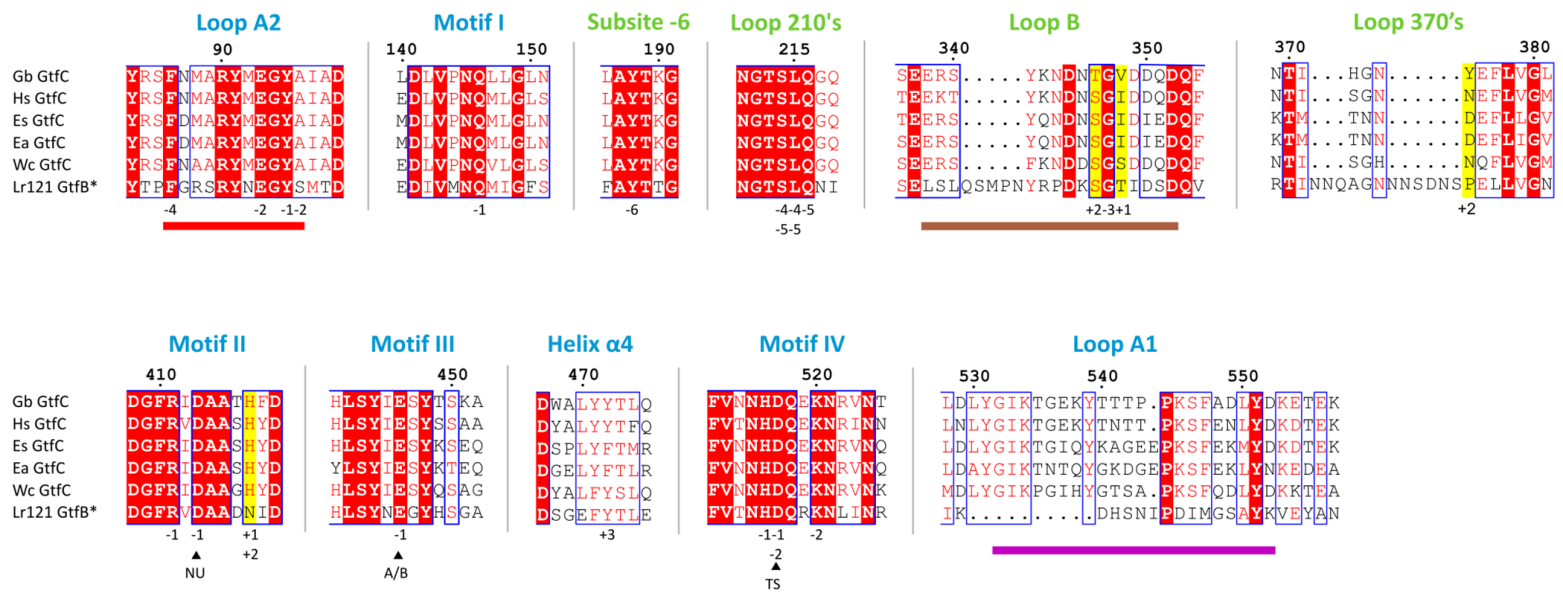
Sequence alignment of selected regions of GH70 4,6-α-glucanotransferases: GtfC-type GTs from *Geobacillus* 12AMOR1 (GbGtfC; this study), *Heyndrickxia sporothermodurans* (HsGtfC),^52^*Exiguobacterium sibiricum* 255–15 (EsGtfC),^18^*Exiguobacterium acetylicum* DSM1 (EaGtfC),^19^*Weissella confusa* (WcGtfC), and a representative GtfB-type GT from *Limosilactobacillus reuteri* 121 (Lr121 GtfB).^25^ Blue headings comprise sections from domain A; green headings are those in domain B. Before alignment, the Lr121 GtfB sequence was manually rearranged (indicated by a *) to match the non-permuted domain organization of GtfC enzymes. The 370s loop was manually aligned based on structural superposition between GbGtfC and Lr121 GtfB. Residue numbering is from GbGtfC. Below the alignment, the subsites with which the respective residues potentially interact are shown, based on the model for donor substrate binding; the four positions near subsites +1 and +2 that vary are indicated with yellow background. The bars below the alignment represent loops A1, A2, and B near the active site; their colors match those in Figure 2b. The three catalytic residues are indicated (NU = nucleophile, A/B = acid/base, TS = transition state stabilizing residue).

The GbGtfC crystal structure is the first representative of the GH70 GtfC α-GT subfamily. Overall, the architecture of its binding groove closely resembles that of the 4,6-α-GT Lr121 GtfB, more than that of α-amylases: while the latter features a fully open binding groove, in GbGtfC, the presence of the two long loops A1 (residues 532–552) and B (residues 338–352) near the binding groove results in a tunnel-like architecture that encompasses donor subsites −2 and −3 (Figure 2b), similar to the situation in Lr121 GtfB.^25^ Alignment of these loops (Figure 3) reveals that their sequences differ significantly from those in Lr121 GtfB and that a shorter loop B is “compensated” by a longer loop A1. The third loop A2 (residues 86–96) lies beneath the binding groove and is highly conserved; it has a similar architecture as in Lr121 GtfB. The tunneled architecture of the binding groove of GbGtfC resembles that of the majority of putative GtfB enzymes^26^ and is in agreement with the fact that GbGtfC products are linear.^22^ As proposed earlier,^25^ the presence of the tunnel may contribute to processivity of the transglycosylation by keeping intermediate products bound to the enzyme; a shift in the direction of the donor side of the binding groove was proposed to explain the observed range of products with consecutive α-1,6 linkages. A different explanation was recently proposed by Yang et al.^50^ stating that intermediate products instead shift toward the acceptor side of the binding groove, keeping intact the hydrogen bond interaction between the 6-OH of the sugar in subsite −1 and a conserved glutamine.

### Product Specificity

**Figure 4:**
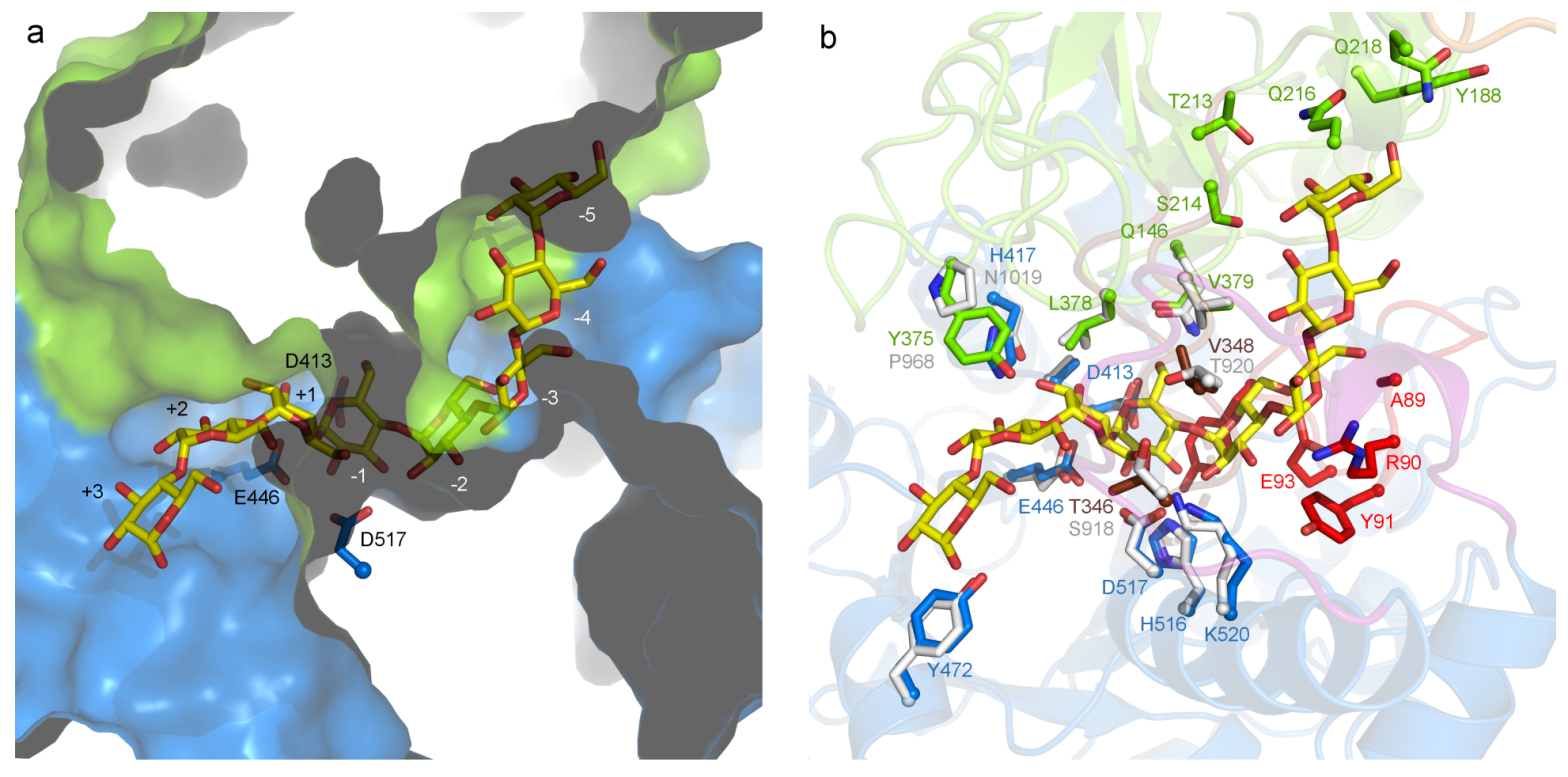
(a) Model of a possible donor substrate, maltooctaose (G8), in the active site groove of GbGtfC with the enzyme represented as a surface; domain A is colored blue and domain B is colored green. The three catalytic residues are shown as sticks. Part of the binding groove features a tunnel spanning at least subsites −2 and −3. (b) The same G8 model, with surrounding residues shown as sticks; residues are colored according to domain (blue = domain A, green = domain B) or loop name (red = loop A2, brown = loop B). Some of the corresponding residues in Lr121 GtfB are shown with gray carbon atoms; in particular, Y375, H417, and T346 (GbGtfC) are close to acceptor subsites +1 and +2 and are replaced by P968, N1019, and S918 from Lr121 GtfB, respectively.

Given the observed structural similarity in the binding groove between GbGtfC and Lr121 GtfB, it is intriguing that Lr121 GtfB (and other GtfBs) synthesize products with consecutive α-1,6 linkages, whereas GbGtfC forms alternating α-1,4/α-1,6 linkages. In fact, GbGtfC so far is the only biochemically characterized GtfC-type 4,6-α-GT displaying this specificity; understanding this unique property is important regarding its application in starch modification.^22,51^ We therefore compared the 3D structures of GbGtfC (this study) and Lr121 GtfB^25^ and used them to perform molecular docking with donor and acceptor substrates. The active site of GbGtfC seems more constrained around subsites +1/+2 than in Lr121 GtfB (Figure S5a); moreover, while the residues surrounding donor subsites are largely conserved, GbGtfC differs at four positions near acceptor binding subsites (Figure 3 and 4b). Residues H417 (motif II) and Y375 (370s loop) belong to the variable set of residues that have been suggested to affect product specificity in GtfB-type α-GTs.^26^ Residue Y375 of GbGtfC is close to subsite +2 and may provide an aromatic stacking platform or a hydrogen bond; for the corresponding P968 of Lr121 GtfB, this is not the case. The larger side chain of Y375 also results in a more constrained acceptor binding space in GbGtfC. Next to Y375 lies H417, near acceptor subsite +1. Mutation of the corresponding N1019 in Lr121 GtfB to histidine significantly changed the linkage ratio (α-1,4/α-1,6) of the products synthesized from amylose.^25^ The third and fourth non-conserved positions, T346 and V348 from loop B, locate at the opposite side of the subsite +1 sugar unit; they are replaced by S918 and T920 in Lr121 GtfB. Together, while the four positions are largely conserved in a subset of 233 GtfBs that likely feature a tunnel (Figure S4), the 63 putative GtfCs have a different and less conserved set. Notably, GbGtfC is unique among GtfCs with Y375 replacing D or N or K and the T346/V348 pair replacing mostly S/I or S/S. This suggests that Y375, T346, and V348 of GbGtfC contribute to its unique product specificity. Supporting evidence comes from a recent study with *H. sporothermodurans* GtfC^52^ postulating that mutation of the corresponding S345/I347 to T/V resulted in products with alternating α-1,6/α-1,4 linkages rather than consecutive α-1,6 linkages.

**Figure 5:**
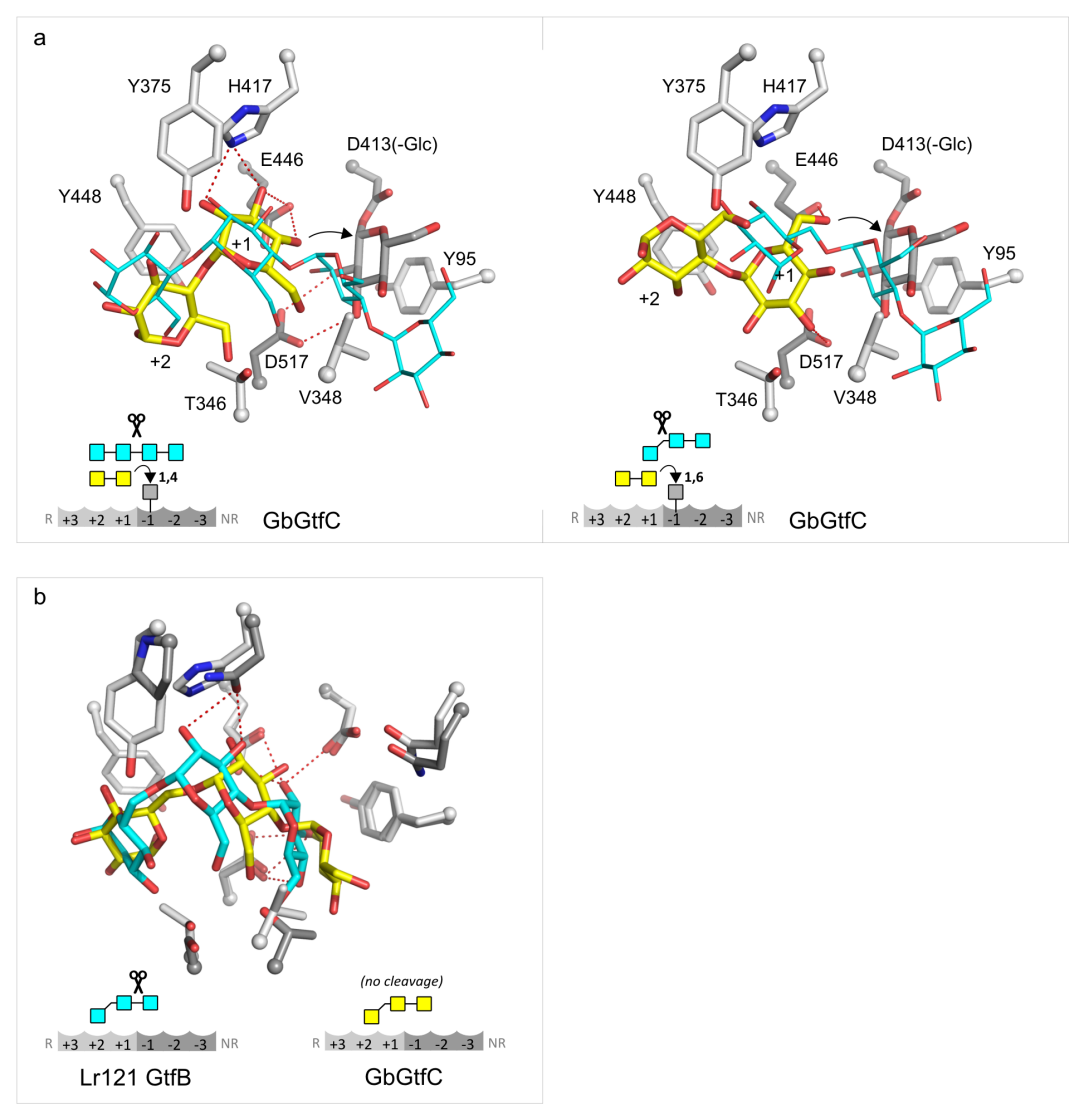
(a) Docking experiments comparing donor and acceptor reactions regarding the +1 sugar unit, shown here for GbGtfC (similar observations were made for Lr121 GtfB). The left panel shows that, for a maltose α-1,4-reacting acceptor (yellow sticks), the conformation of the +1 glucosyl does not differ much from that of a maltotetraose donor (cyan lines). In contrast, for α-1,6-specific donor and acceptor reactions, the +1 sugar unit assumes very different orientations, as is shown for a maltose acceptor (yellow sticks) and 6′*O*-α-maltotriosyl-glucose donor (cyan lines) (right panel). (b) Docking of isopanose in GbGtfC (yellow and light gray carbon atoms for ligand and surrounding residues, respectively) and Lr121 GtfB (cyan and dark gray carbon atoms, respectively). In contrast to the situation in Lr121 GtfB, the trisaccharide assumes a conformation unlikely to be α-1,4 cleaved by GbGtfC.

It was proposed earlier that α-1,4/α-1,6 alternating end products of GbGtfC can be explained by an α-1,6 transglycosylation preference for maltosyl rather than glucosyl moieties, supported by the accumulation of maltose and hardly any glucose upon incubation of the enzyme with amylose V.^22^ However, the synthesis of α-glucans by 4,6-α-GTs proceeds through many cleavage and transfer steps. To understand how the final product spectrum is obtained would require a systematic analysis of every possible reaction for each possible donor or acceptor substrate. Indeed, our docking experiments suggested that the situation is more complicated than can be explained by a single transglycosylation preference. Nevertheless, the docking experiments with GbGtfC and Lr121 GtfB (methods and results described in the Supporting Information and Figure S5) did allow us to derive some principles that agree with the experimentally observed end products of either enzyme.^22,25^ First, a general and rather unexpected observation was that, for both enzymes, donor and acceptor reactions do not seem to be restricted to α-1,4-resp. α-1,6-specificity, but also can occur with α-1,6- resp. α-1,4-specificity. Yet, α-1,6 transglycosylations become dominant over α-1,4 transglycosylations, because (intermediate) products of the latter can easily “react back” because the glucosyl moiety in subsite +1 hardly requires a change in conformation to act in a subsequent donor reaction (Figure 5a). In contrast, α-1,6-transglycosylation products do not react back as donors, as a large reorientation would be needed for the subsite +1 glucosyl moiety to do so. Second, we found that both enzymes are able to transfer glucosyl as well as maltosyl moieties, but GbGtfC seems to be less efficient in cleaving the non-reducing end (NR) terminal α-1,4 linkage from maltosyl-ending intermediate products (Figure 5b). For example, in a docking scenario with isopanose in GbGtfC, the α-1,4 linkage did not assume a favorable position for cleavage while the α-1,6 linkage did (Figure S5b). The result is that, with GbGtfC, intermediate products with NR maltosyl ends “survive”, and these are easily elongated by α-1,6-transglycosylation, favoring the formation of alternating glucan products. The experimentally observed maltose in the reaction pool of GbGtfC^22^ likely results from a more efficient α-1,4-transglycosylation of glucose than in Lr121 GtfB. Finally, the docking results suggest that the described differences between GbGtfC and Lr121 GtfB relate to interactions of donor/acceptor substrates in subsites +1 and +2 with the non-conserved residues described above (Table S3), further supporting the role of these residues in determining the unique product specificity of GbGtfC.

### AlphaFold Model of Full-Length GbGtfC and Other GtfC Enzymes

**Figure 6:**
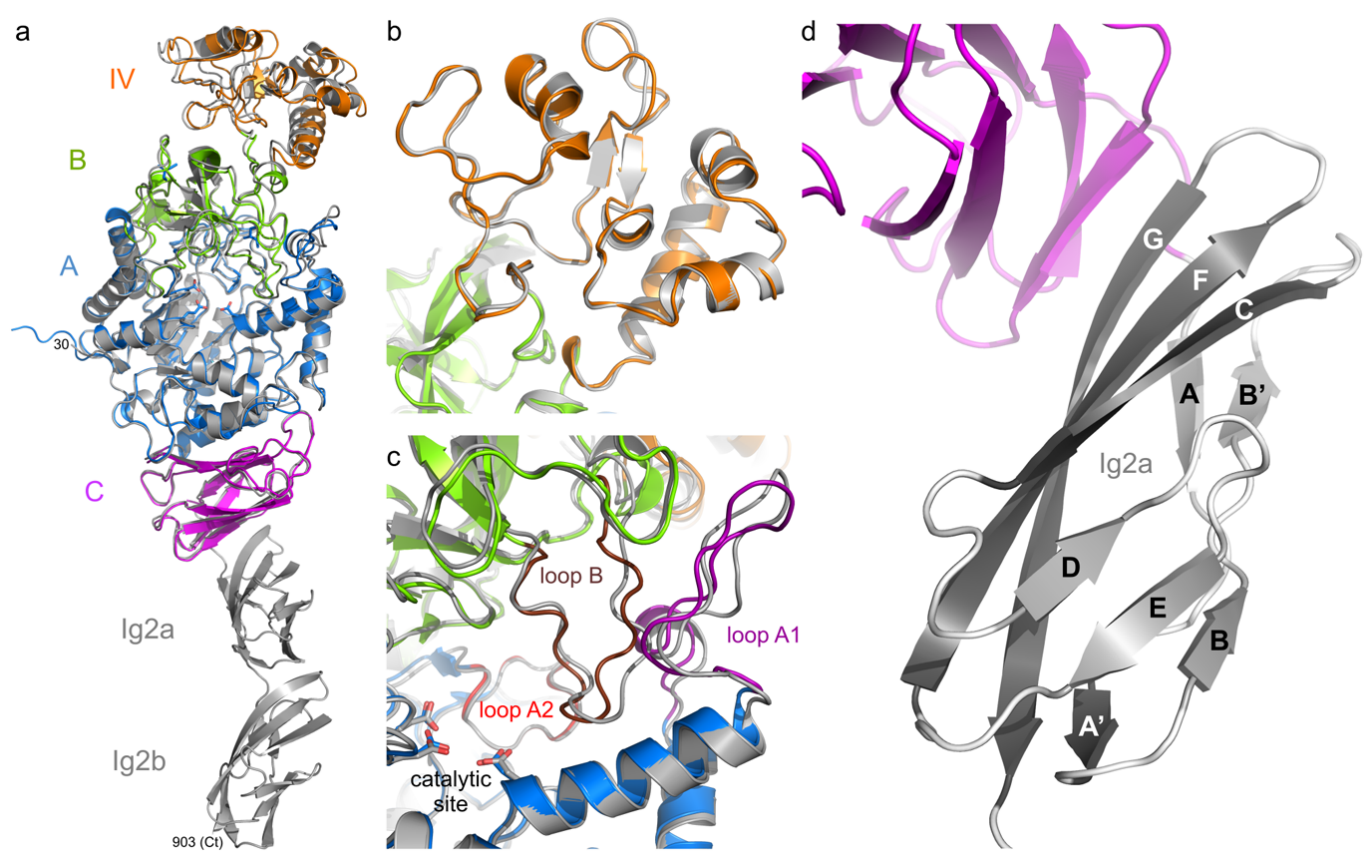
AlphaFold model of GbGtfC (gray) superposed on the GbGtfC-ΔC crystal structure (colored domains); the 29 N-terminal residues of the AlphaFold model were omitted while the C-terminal Ig2 domains extend away from domain C. (a) Overall superposition with RMSD = 0.79 Å. (b) Superposition based on domain IV (residues 223–332), with RMSD = 0.55 Å. (c) Loop regions near the active site. (d) Topology of the Ig2a domain of the AlphaFold model with the β-strands labeled; the Ig2b domain (see a) has the same topology.

The average per-residue confidence score (pLDDT) of the highest ranked AlphaFold model of GbGtfC was 92.6. The N-terminal 32 residues of GbGtfC correspond to the signal peptide and expectedly showed significantly lower pLDDT scores (Figure S6a); omitting these residues improved the average pLDDT to 94.8 (Table S1), indicating a highly reliable model. The AlphaFold model superposed well with the crystal structure (RMSD = 0.79 Å for 591 Cα atoms), even for most of the loop regions (Figure 6a); nevertheless, some differences were observed. First, domain IV has a slightly different orientation relative to the core of the enzyme (Figure 6a), supporting the notion that this domain may be slightly flexible around the hinge formed by the two loops connecting it to domain B. On its own, the modeled domain IV superimposes well with that in the crystal structure (Figure 6b) and includes the segments that showed poorly defined electron density. The second most obvious differences between the modeled and experimental structure occur in the loop regions near the active site (Figure 6c). The AlphaFold models show slightly different conformations of loops A1 and B, with shifts up to 3.6 Å with respect to the crystal structure, but the general course of the loops is the same. In the active site region, almost all side chains were modeled with the same rotamer as that of the crystal structure; exceptions are H372, Y375 and L378 (not shown).

The AlphaFold model of GbGtfC also includes the C-terminal ∼165 residues that are absent in the crystallized construct; as predicted previously,^22^ they form two bacterial Ig-like type 2 domains (Ig2), which connect to domain C *via* a short loop (residues 734–738) (Figure 6a). Although the high pLDDT scores for the Ig2 domains of GbGtfC (Figure S6a) indicate reliable modeling of their fold, the relative orientation of these domains is modeled with less confidence, especially regarding the C-terminal Ig2 domain. Domain Ig2a (residues 739–823) and domain Ig2b (residues 824–903) share low sequence identity (26.2%) but have the same immunoglobulin fold; they can be superimposed giving an RMSD of 0.74 Å. Both domains contain nine β-strands and form two opposing, mostly antiparallel β-sheets (Figure 6d). However, the first two β-strands (A and B) can be considered interrupted, and this results in subsheets composed of A–B’, B–E–D, and A’–G–F–C.

The BLASTp results indicate that on a residue level GbGtfC is rather unique among GtfC subfamily enzymes: it is the only enzyme from a *Geobacillus* species, and the closest homologues in terms of sequence (from *H. sporothermodurans*) show 76.3% sequence identity. Some of its residues near the binding groove are different from most GtfC sequences (see above). This raised the question how representative the GbGtfC 3D structure is for the GtfC subfamily of 4,6-α-GTs. We therefore constructed AlphaFold models of four other GtfC-type GTs (Table S1), three of which were characterized as 4,6-α-GTs synthesizing linear isomalto/maltooligosaccharides with consecutive α-1,6 linkages. The AlphaFold models showed comparable pLDDT scores and very similar folds (Figure S7a), reflected in low RMSD values of 0.54–0.72 Å upon Cα superposition with GbGtfC. Notably, the high structural conservation includes not only the core domains A, B, and C but also domain IV. Near the active site region, loops A1, A2, and B have somewhat lower pLDDT scores (not shown). Although there are slight differences in position with differences up to 3.8 Å (in the tip of loop A1), these loops have the same architecture as in GbGtfC and form a tunnel at the donor side of the binding groove (Figure S7b). We thus suggest that, although *Geobacillus* 12AMOR1 GbGtfC has some unique features near the active site, the 3D structure of the core domains of this enzyme represents the whole GtfC subfamily, at least for the 63 sequences found so far.

Like GbGtfC, the C-terminal domains of the GtfC from *H. sporothermodurans*, *E. acetylicum*, and *E. sibiricum* 255-15 feature two Ig2 domains; for the latter, this was already predicted in an earlier study.^18^ Ig2 domains occur in various bacterial and phage surface proteins and have been proposed to play a role in cell surface adhesion or carbohydrate binding.^53^ For GtfCs, this remains to be investigated, but since these enzymes are extracellular and process carbohydrates, such functions seem to be possible. On the other hand, the predicted structure of the *W. coagulans* DSM1 GtfC features three C-terminal SRC Homology 3 (SH3) domains (Figure S7a) of about 60 residues each; SH3 domains are thought to mediate protein–protein interactions.^54^ Variations in the length of the C-terminal parts of the GtfC sequences found by the BLASTp search (see below) suggests that the type and the number of copies of the C-terminal domains could be related to the bacterial species and its specific natural environment.

### Phylogenetic Relations and Evolutionary Aspects

A BLASTp search with the *Geobacillus* 12AMOR1 GtfC (GbGtfC) sequence yielded a total of 102 putative non-permuted bacterial sequences containing the four conserved GH70 motifs in the order I–II–III–IV (Table S2). All sequences originate from non-LAB species, but based on their sequence alignment they could be divided in two groups. The first group contains 63 hits, more than double the number of sequences identified in 2018^2^ and shows sequence identities of 52.9–76.3% with GbGtfC. The enzymes within this group originate mainly from Gram-positive soil or marine bacteria such as *Weizmannia coagulans* or *Exiguobacterium* species; for example, the earlier characterized GtfCs from *Exiguobacterium sibiricum* 255-15^18^ and *Weizmannia coagulans* DSM1^20^ belong to this group. Most sequences have a length of around 900 residues and share high sequence similarity, suggesting that they are GtfC-type α-glucanotransferases constituting a similar domain organization with the three core domains (A, B, and C), an inserted domain IV, and extra C-terminal domains. The second group, containing the remaining 39 sequences, showed lower overall sequence identities (40.4–49.9% to GbGtfC) and originate mostly from Gram-negative bacteria such as *Azotobacter chroococcum* (a plant-associated nitrogen-fixing species) or *Burkholderia* (animal/plant pathogen). Including the previously characterized enzymes from *Azotobacter chroococcum* NCIMB 8003^24^ and from the Gram-positive *Paenibacillus beijingensis* DSM 24997,^23^ this group represents putative GtfD-type α-glucanotransferases. In general, the sequences in this group are shorter at the C-terminal end, suggesting that they do not feature Ig-like domains.

A more detailed analysis of the sequence alignment of the GH70 motifs, loops A1, A2, B, and the 370s loop within the GtfC group revealed that they are highly conserved regarding residue type (selected enzymes from this group are shown in Figure 3) as well as loop length (Table S2). The *Geobacillus* 12AMOR1 GtfC sequence is rather unique in these regions (note that it is the only *Geobacillus* entry found). Nevertheless, the alignment strongly suggests that all 63 putative GtfC-type α-GTs found so far feature a tunneled binding groove, prefer mostly linear starch substrates, and synthesize linear α-glucan products; this is also supported by the AlphaFold models of selected GtfCs (see above). Whether GbGtfC is the only GtfC synthesizing products with alternating α-1,4/α-1,6 linkages remains to be investigated. A detailed biochemical characterization of more GtfC-type GTs and their products is needed to confirm this.

**Figure 7:**
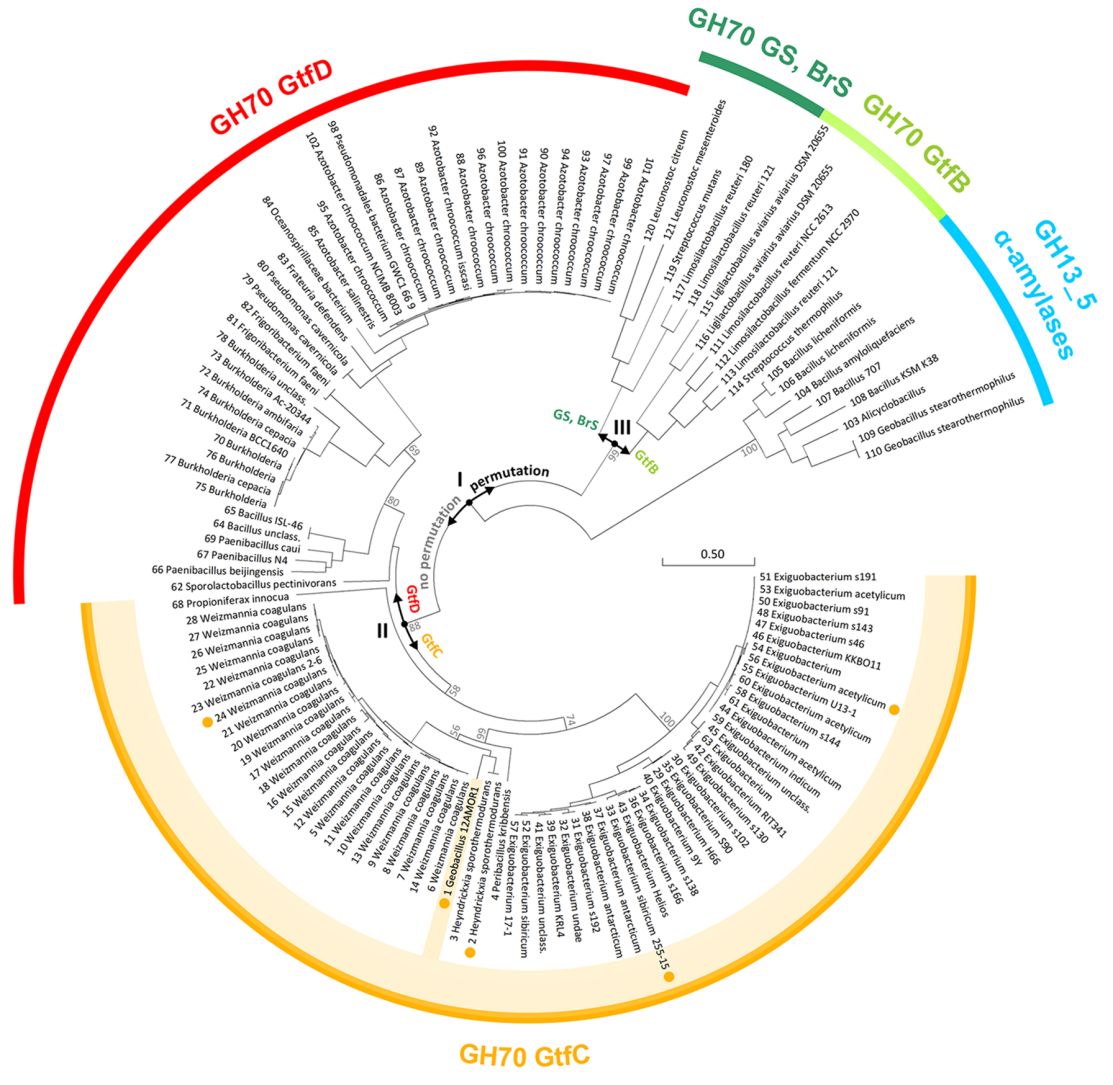
Unrooted phylogenetic tree calculated from the 121 GH70 and GH13 amino acid sequences. GH70 contains several subfamilies: glucansucrases (GS)/branching sucrases (Brs), GtfB-, GtfC-, and GtfD-type α-GTs, indicated by different colors. The number preceding each sequence corresponds to the numbering in Table S2. GbGtfC (this study) is highlighted; sequences for which an AlphaFold model has been calculated are indicated with a yellow dot. Important evolutionary branch separation events are indicated (I, II, and III).

A phylogenetic tree generated from the extended alignment of GH70 and GH13_5 enzymes (Figure 7) sheds more light on the GH13/GH70 evolutionary pathways originally conceived by Vujičić-Žagar et al.^55^ and later extended/refined by Gangoiti et al.^18^ A clear distinction is seen between the GH13_5 α-amylases that degrade but not transglycosylate starch substrates and the GH70 enzymes that acquired α-1,6 transglycosylation capabilities. Importantly, for the GH70 sequences, three bifurcation points (I, II, and III) are apparent (Figure 7). Point I signifies the distinction between non-permuted and permuted GH70 enzymes. On one hand, in non-LAB species, the enzymes remained non-permuted, and later evolved differently in Gram-positive (GtfC) or (mostly) Gram-negative (GtfD) enzymes (point II): while the GtfC-type enzymes acquired extra C-terminal domains and kept the tunnel-like architecture, the GtfD-type enzymes seem to have evolved to feature shorter loops A1 (Table S2) likely related to their reaction specificity involving more branched substrates and products.^23,24^ On the other hand, in LAB species, permutation did take place (*via* gene duplication) (Figure 7); a later bifurcation (point III) signifies that part of the enzymes changed their substrate specificity from starch (GtfB) to sucrose (glucansucrases, branching sucrases) by further adapting their active site architecture.^25^

**Figure 8:**
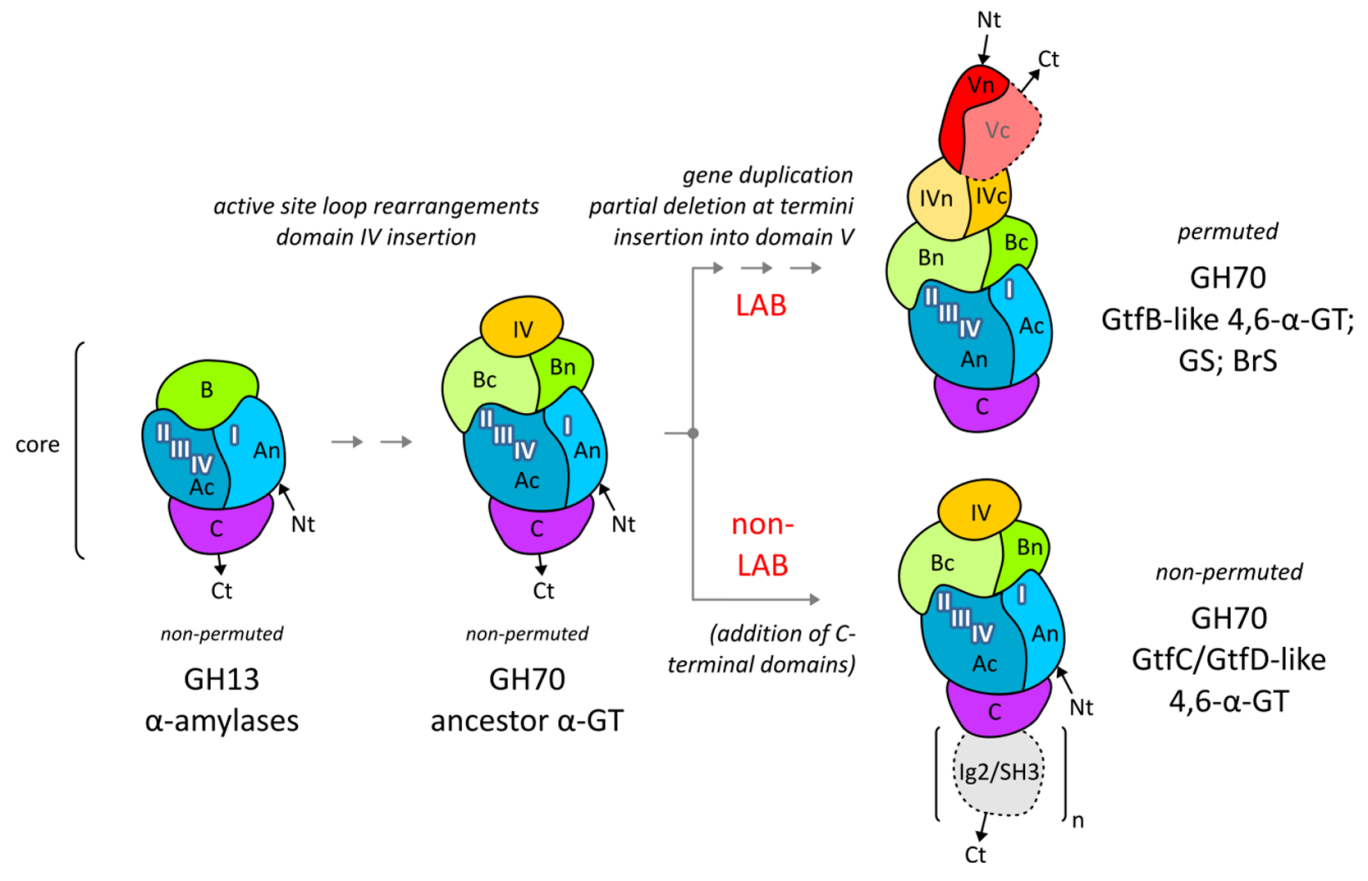
Evolutionary pathway depicting the domain organization and permutation in GH13 and GH70 enzymes, partly based on earlier findings.^18,55^ The core domains A, B, and C are present throughout; N- and C-termini are indicated with Nt and Ct, respectively. GH13 α-amylases, appearing in all kingdoms of life, acquired transglycosylation specificity by changing structural elements around the active site, while the additional insertion of domain IV into domain B resulted in a (still non-permuted) GH70 ancestor α-GT. From such an ancestor (likely corresponding to point I in Figure 6), two “branches” evolved. The first branch evolved in non-LAB species: GH70 GtfC- and GtfD-type 4,6-α-glucanotransferase enzymes (4,6-α-GT) remained non-permuted, featuring the same single-segment domain IV, and acquiring additional C-terminal Ig2- or SH3-type domains. In the second branch, evolving in LAB species, the GH70 GtfB-type, glucansucrase (GS) and branching sucrase (BrS) enzymes became circularly permuted, with domain IV consisting of two segments far apart in sequence. The enzymes in this branch acquired different auxiliary domains at their N- and/or C-termini, again far apart in sequence. Notably, as the GbGtfC crystal structure shows, the structure of the core domains in the non-LAB and LAB branches is remarkably similar, especially in the active site region. In all (sub)families, domain A contains the four homology motifs I–IV; circular permutation in GH70 enzymes changes their order such that motif I is placed C-terminal of motifs II–III–IV; thus, the order changes from I–II–III–IV to II–III–IV–I.

Notably, despite the absence of permutation and despite a different domain composition, the GtfC- and GtfD-clades are phylogenetically closer to other GH70 enzymes (GtfB-type α-GTs, glucansucrases, and branching sucrases) than they are to the GH13_5 α-amylases. The GbGtfC-ΔC crystal structure (as well as the AlphaFold models of other GtfC enzymes) clearly confirms this, showing the high structural similarity with GtfB enzymes, but differing from the GH13_5 α-amylases, which have a more open active site groove and lack certain structural elements in the core domains (e.g., a two-helix/loop insertion between β-strands 7 and 8, as well as the long loops A1 and B). Thus, the high structural similarity between GtfC and GtfB-type α-GTs shows that the gene duplication step occurring in LAB did not lead to large structural changes in the core domains, consistent with their shared substrate and reaction specificity (α-1,4 cleavage followed by α-1,6-transglycosylation of starch-like compounds). This also suggests that the changes that were necessary to acquire α-1,6 transglycosylation specificity, as well as the insertion of domain IV, took place before the division between LAB and non-LAB (bifurcation point I), likely in bacterial α-amylase enzymes and leading to an ancestor α-GT enzyme (Figure 8). The role of domain IV in GH70 enzymes and why it was inserted is unclear; while there are examples of starch-targeting GH13 α-amylases with a carbohydrate binding domain (CBM) inserted in domain B,^56^ in GbGtfC (and other GH70 enzymes), domain IV structurally does not resemble a CBM domain and did not reveal carbohydrate binding sites. Finally, the phylogenetic tree shows that within the GtfC clade, the *Geobacillus* 12AMOR1 GtfC is in a rather unique position, perhaps related to the observed differences in residues surrounding the binding groove as described above.

## Supporting Information Available

Figures of elution profile of three size exclusion chromatography runs, crystal structure of GbGtfC-ΔC, stereo figures of the GbGtfC crystal structure and electron density, sequence logos of four non-conserved residues, selected docking results for donor and acceptor reactions in GbGtfC and Lr121 GtfB, AlphaFold model of GbGtfC, and comparison of AlphaFold models of GbGtfC and four other GtfC-type GTs, discussion of molecular docking, and tables of AlphaFold models of selected GtfC-type GTs with their relative sequence identity, list of sequences used for the alignment of 121 GH70/GH13 enzymes using the GbGtfC sequence as reference, correlation between reactivity and subsite +1/+2 glucosyl interactions, and references (PDF)

## References

[ref1] Gangoiti J.; Corwin S. F.; Lamothe L. M.; Vafiadi C.; Hamaker B. R.; Dijkhuizen L. Synthesis of novel α-glucans with potential health benefits through controlled glucose release in the human gastrointestinal tract. Crit. Rev. Food Sci. Nutr. 2020, 60, 123–146. 10.1080/10408398.2018.1516621. 30525940

[ref2] Gangoiti J.; Pijning T.; Dijkhuizen L. Biotechnological potential of novel glycoside hydrolase family 70 enzymes synthesizing α-glucans from starch and sucrose. Biotechnol. Adv. 2018, 36, 196–207. 10.1016/j.biotechadv.2017.11.001. 29133008

[ref3] Te Poele E. M.; Corwin S. G.; Hamaker B. R.; Lamothe L. M.; Vafiadi C.; Dijkhuizen L. Development of slowly digestible starch derived α-glucans with 4,6-α-glucanotransferase and branching sucrase enzymes. J. Agric. Food Chem. 2020, 68, 6664–6671. 10.1021/acs.jafc.0c01465. 32437608 PMC7304062

[ref4] Gu F.; Borewicz K.; Richter B.; van der Zaal P. H.; Smidt H.; Buwalda P. L.; Schols H. A. *In Vitro* fermentation behavior of isomalto/malto-polysaccharides using human fecal inoculum indicates prebiotic potential. Mol. Nutr. Food Res. 2018, 62, 1800232 10.1002/mnfr.201800232. 29710405 PMC6033187

[ref5] Jurášková D.; Ribeiro S. C.; Silva C. C. G. Exopolysaccharides produced by lactic acid bacteria: from biosynthesis to health-promoting properties. Foods 2022, 11, 156 10.3390/foods11020156. 35053888 PMC8774684

[ref6] Miao M.; Jiang B.; Jin Z.; BeMiller J. N. Microbial starch-converting enzymes: Recent insights and perspectives. Compr. Rev. Food Sci. Food Saf. 2018, 17, 1238–1260. 10.1111/1541-4337.12381. 33350152

[ref7] Leemhuis H.; Dobruchowska J. M.; Ebbelaar M.; Faber F.; Buwalda P. L.; van der Maarel M. J.; Kamerling J. P.; Dijkhuizen L. Isomalto/malto-polysaccharide, a novel soluble dietary fiber made via enzymatic conversion of starch. J. Agric. Food Chem. 2014, 62, 12034–12044. 10.1021/jf503970a. 25412115

[ref8] Meng X.; Gangoiti J.; Bai Y.; Pijning T.; Van Leeuwen S. S.; Dijkhuizen L. Structure-function relationships of family GH70 glucansucrase and 4,6-α-glucanotransferase enzymes, and their evolutionary relationships with family GH13 enzymes. Cell. Mol. Life Sci. 2016, 73, 2681–2706. 10.1007/s00018-016-2245-7. 27155661 PMC4919382

[ref9] Li X.; Fei T.; Wang Y.; Zhao Y.; Pan Y.; Li D. Wheat starch with low retrogradation properties produced by modification of the GtfB enzyme 4,6-α-glucanotransferase from *Streptococcus thermophilus*. J. Agric. Food Chem. 2018, 66, 3891–3898. 10.1021/acs.jafc.8b00550. 29582651

[ref10] van der Zaal P. H.; Schols H. A.; Bitter J. H.; Buwalda P. L. Isomalto/malto-polysaccharide structure in relation to the structural properties of starch substrates. Carbohydr. Polym. 2018, 185, 179–186. 10.1016/j.carbpol.2017.11.072. 29421055

[ref11] Kralj S.; Grijpstra P.; van Leeuwen S. S.; Leemhuis H.; Dobruchowska J. M.; van der Kaaij R. M.; Malik A.; Oetari A.; Kamerling J. P.; Dijkhuizen L. 4,6-α-Glucanotransferase, a novel enzyme that structurally and functionally provides an evolutionary link between Glycoside Hydrolase enzyme families 13 and 70. Appl. Environ. Microbiol. 2011, 77, 8154–8163. 10.1128/AEM.05735-11. 21948833 PMC3209003

[ref12] Leemhuis H.; Dijkman W. P.; Dobruchowska J. M.; Pijning T.; Grijpstra P.; Kralj S.; Kamerling J. P.; Dijkhuizen L. 4,6-α-Glucanotransferase activity occurs more widespread in *Lactobacillus* strains and constitutes a separate GH70 subfamily. Appl. Microbiol. Biotechnol. 2013, 97, 181–193. 10.1007/s00253-012-3943-1. 22361861 PMC3536977

[ref13] Gangoiti J.; van Leeuwen S. S.; Gerwig G. J.; Duboux S.; Vafiadi C.; Pijning T.; Dijkhuizen L. 4,3-α-Glucanotransferase, a novel reaction specificity in Glycoside Hydrolase family 70 and clan GH-H. Sci. Rep. 2017, 7, 39761 10.1038/srep39761. 28059108 PMC5216370

[ref14] Gangoiti J.; van Leeuwen S. S.; Meng X.; Duboux S.; Vafiadi C.; Pijning T.; Dijkhuizen L. Mining novel starch-converting Glycoside Hydrolase 70 enzymes from the Nestlé Culture Collection genome database: The *Lactobacillus reuteri* NCC 2613 GtfB. Sci. Rep. 2017, 7, 9947 10.1038/s41598-017-07190-z. 28855510 PMC5577214

[ref15] Meng X.; Gangoiti J.; de Kok N.; van Leeuwen S. S.; Pijning T.; Dijkhuizen L. Biochemical characterization of two GH70 family 4,6-α-glucanotransferases with distinct product specificity from *Lactobacillus aviarius* subsp. *aviarius* DSM 20655. Food Chem. 2018, 253, 236–246. 10.1016/j.foodchem.2018.01.154. 29502827

[ref16] İspirli H.; Şimşek Ö.; Skory C.; Sağdıç O.; Dertli E. Characterization of a 4,6-α-glucanotransferase from *Lactobacillus reuteri* E81 and production of malto-oligosaccharides with immune-modulatory roles. Int. J. Biol. Macromol. 2019, 124, 1213–1219. 10.1016/j.ijbiomac.2018.12.050. 30529203

[ref17] Yang W.; Sheng L.; Chen S.; Wang L.; Su L.; Wu J. Characterization of a new 4,6-α-glucanotransferase from *Limosilactobacillus fermentum* NCC 3057 with ability of synthesizing low molecular mass isomalto-/maltopolysaccharide. Food Bioscience 2022, 46, 101514 10.1016/j.fbio.2021.101514.

[ref18] Gangoiti J.; Pijning T.; Dijkhuizen L. The *Exiguobacterium sibiricum* 255–15 GtfC enzyme represents a novel Glycoside Hydrolase 70 subfamily of 4,6-α-glucanotransferase enzymes. Appl. Environ. Microbiol. 2016, 82, 756–766. 10.1128/AEM.03420-15. 26590275 PMC4711130

[ref19] Kralj S. Compositions and methods comprising the use of *Exiguobacterium acetylicum* and *Bacillus coagulans* α-glucanotransferase enzymes. US11072783B2, 2017.

[ref20] Xiang G.; Buwalda P. L.; van der Maarel M. J. E. C.; Leemhuis H. The thermostable 4,6-α-glucanotransferase of *Bacillus coagulans* DSM 1 synthesizes isomaltooligosaccharides. Amylase 2021, 5, 13–22. 10.1515/amylase-2021-0002.

[ref21] Wissuwa J.; Stokke R.; Fedøy A. E.; Lian K.; Smalås A. O.; Steen I. H. Isolation and complete genome sequence of the thermophilic *Geobacillus* sp. 12AMOR1 from an Arctic deep-sea hydrothermal vent site. Stand. Genomic Sci. 2016, 11, 16 10.1186/s40793-016-0137-y. 26913091 PMC4765119

[ref22] Te Poele E. M.; van der Hoek S. E.; Chatziioannou A. C.; Gerwig G. J.; Duisterwinkel W. J.; Oudhuis L.A.A.C.M.; Gangoiti J.; Dijkhuizen L.; Leemhuis H. GtfC enzyme of *Geobacillus* sp. 12AMOR1 represents a novel thermostable type of GH70 4,6-α-glucanotransferase that synthesizes a linear alternating (α1 → 6)/(α1 → 4) α-glucan and delays bread staling. J. Agric. Food Chem. 2021, 69, 9859–9868. 10.1021/acs.jafc.1c03475. 34427087

[ref23] Gangoiti J.; Lamothe L.; van Leeuwen S. S.; Vafiadi C.; Dijkhuizen L. Characterization of the *Paenibacillus beijingensis* DSM 24997 GtfD and its glucan polymer products representing a new Glycoside Hydrolase 70 subfamily of 4,6-α-glucanotransferase enzymes. PLoS One 2017, 12, e0172622 10.1371/journal.pone.0172622. 28399167 PMC5388325

[ref24] Gangoiti J.; van Leeuwen S. S.; Vafiadi C.; Dijkhuizen L. The Gram-negative bacterium *Azotobacter chroococcum* NCIMB 8003 employs a new Glycoside Hydrolase family 70 4,6-α-glucanotransferase enzyme (GtfD) to synthesize a reuteran like polymer from maltodextrins and starch. Biochim. Biophys. Acta 2016, 1860, 1224–1236. 10.1016/j.bbagen.2016.02.005. 26868718

[ref25] Bai Y.; Gangoiti J.; Dijkstra B. W.; Dijkhuizen L.; Pijning T. Crystal structure of 4,6-α-glucanotransferase supports diet-driven evolution of GH70 enzymes from α-amylases in oral bacteria. Structure 2017, 25, 231–242. 10.1016/j.str.2016.11.023. 28065507

[ref26] Pijning T.; Gangoiti J.; Te Poele E. M.; Börner T.; Dijkhuizen L. Insights into broad-specificity starch modification from the crystal structure of *Limosilactobacillus reuteri* NCC 2613 4,6-α-glucanotransferase GtfB. J. Agric. Food Chem. 2021, 69, 13235–13245. 10.1021/acs.jafc.1c05657. 34708648 PMC8587608

[ref27] MacGregor E. A.; Jespersen H. M.; Svensson B. A circularly permuted α-amylase-type α/β-barrel structure in glucan-synthesizing glucosyltransferases. FEBS Lett. 1996, 378, 263–266. 10.1016/0014-5793(95)01428-4. 8557114

[ref28] Janeček Š.; Svensson B.; MacGregor E. A. α-Amylase: an enzyme specificity found in various families of glycoside hydrolases. Cell. Mol. Life Sci. 2014, 71, 1149–1170. 10.1007/s00018-013-1388-z. 23807207 PMC11114072

[ref29] Kabsch W. XDS. Acta Crystallogr. D Biol. Crystallogr. 2010, 66, 125–132. 10.1107/S0907444909047337. 20124692 PMC2815665

[ref30] McCoy A. J.; Grosse-Kunstleve R. W.; Adams P. D.; Winn M. D.; Storoni L. C.; Read R. J. Phaser crystallographic software. J. Appl. Crystallogr. 2007, 40, 658–674. 10.1107/S0021889807021206. 19461840 PMC2483472

[ref31] Kelley L. A.; Mezulis S.; Yates C. M.; Wass M. N.; Sternberg M. J. The Phyre2 web portal for protein modeling, prediction and analysis. Nat. Protoc. 2015, 10, 845–858. 10.1038/nprot.2015.053. 25950237 PMC5298202

[ref32] Tan T. C.; Mijts B. N.; Swaminathan K.; Patel B. K.; Divne C. Crystal structure of the polyextremophilic α-amylase AmyB from *Halothermothrix orenii*: details of a productive enzyme-substrate complex and an N domain with a role in binding raw starch. J. Mol. Biol. 2008, 378, 852–870. 10.1016/j.jmb.2008.02.041. 18387632

[ref33] Murshudov G. N.; Vagin A. A.; Dodson E. J. Refinement of macromolecular structures by the maximum-likelihood method. Acta Crystallogr. D Biol. Crystallogr. 1997, 53, 240–255. 10.1107/S0907444996012255. 15299926

[ref34] Emsley P.; Lohkamp B.; Scott W. G.; Cowtan K. Features and development of Coot. Acta Crystallogr. D. Struct. Biol. 2010, 66, 486–501. 10.1107/S0907444910007493. PMC2852313 20383002

[ref35] Liebschner D.; Afonine P. V.; Baker M. L.; Bunkóczi G.; Chen V. B.; Croll T. I.; Hintze B.; Hung L. W.; Jain S.; McCoy A. J.; Moriarty N. W.; Oeffner R. D.; Poon B. K.; Prisant M. G.; Read R. J.; Richardson J. S.; Richardson D. C.; Sammito M. D.; Sobolev O. V.; Stockwell D. H.; Terwilliger T. C.; Urzhumtsev A. G.; Videau L. L.; Williams C. J.; Adams P. D. Macromolecular structure determination using X-rays, neutrons and electrons: recent developments in Phenix. Acta Crystallogr. D. Struct. Biol. 2019, 75, 861–877. 10.1107/S2059798319011471. 31588918 PMC6778852

[ref36] Jumper J.; Evans R.; Pritzel A.; Green T.; Figurnov M.; Ronneberger O.; Tunyasuvunakool K.; Bates R.; Žídek A.; Potapenko A.; Bridgland A.; Meyer C.; Kohl S. A. A.; Ballard A. J.; Cowie A.; Romera-Paredes B.; Nikolov S.; Jain R.; Adler J.; Back T.; Petersen S.; Reiman D.; Clancy E.; Zielinski M.; Steinegger M.; Pacholska M.; Berghammer T.; Bodenstein S.; Silver D.; Vinyals O.; Senior A. W.; Kavukcuoglu K.; Kohli P.; Hassabis D. Highly accurate protein structure prediction with AlphaFold. Nature 2021, 596, 583–589. 10.1038/s41586-021-03819-2. 34265844 PMC8371605

[ref37] Varadi M.; Anyango S.; Deshpande M.; Nair S.; Natassia C.; Yordanova G.; Yuan D.; Stroe O.; Wood G.; Laydon A.; Žídek A.; Green T.; Tunyasuvunakool K.; Petersen S.; Jumper J.; Clancy E.; Green R.; Vora A.; Lutfi M.; Figurnov M.; Cowie A.; Hobbs N.; Kohli P.; Kleywegt G.; Birney E.; Hassabis D.; Velankar S. AlphaFold protein structure database: massively expanding the structural coverage of protein-sequence space with high-accuracy models. Nucleic Acids Res. 2022, 50, D439–D444. 10.1093/nar/gkab1061. 34791371 PMC8728224

[ref38] Touw W. G.; Baakman C.; Black J.; te Beek T. A.; Krieger E.; Joosten R. P.; Vriend G. A series of PDB-related databanks for everyday needs. Nucleic Acids Res. 2015, 43, D364–8. 10.1093/nar/gku1028. 25352545 PMC4383885

[ref39] Krissinel E.; Henrick K. Secondary-structure matching (SSM), a new tool for fast protein structure alignment in three dimensions. Acta Crystallogr. D Biol. Crystallogr. 2004, 60, 2256–2268. 10.1107/S0907444904026460. 15572779

[ref40] Robert X.; Gouet P. Deciphering key features in protein structures with the new ENDscript server. Nucleic Acids Res. 2014, 42, W320–4. 10.1093/nar/gku316. 24753421 PMC4086106

[ref41] Bohne A.; Lang E.; von der Lieth C. W. SWEET - WWW-based rapid 3D construction of oligo- and polysaccharides. Bioinformatics 1999, 15, 767–768. 10.1093/bioinformatics/15.9.767. 10498779

[ref42] Morris G. M.; Huey R.; Lindstrom W.; Sanner M. F.; Belew R. K.; Goodsell D. S.; Olson A. J. AutoDock4 and AutoDockTools4: Automated docking with selective receptor flexibility. J. Comput. Chem. 2009, 30, 2785–2791. 10.1002/jcc.21256. 19399780 PMC2760638

[ref43] Nivedha A. K.; Thieker D. F.; Makeneni S.; Hu H.; Woods R. J. Vina-Carb: Improving glycosidic angles during carbohydrate docking. J. Chem. Theory Comput. 2016, 12, 892–901. 10.1021/acs.jctc.5b00834. 26744922 PMC5140039

[ref44] Edgar R. C. MUSCLE: multiple sequence alignment with high accuracy and high throughput. Nucleic Acids Res. 2004, 32, 1792–1797. 10.1093/nar/gkh340. 15034147 PMC390337

[ref45] Waterhouse A. M.; Procter J. B.; Martin D. M.; Clamp M.; Barton G. J. Jalview Version 2 - a multiple sequence alignment editor and analysis workbench. Bioinformatics 2009, 25, 1189–1191. 10.1093/bioinformatics/btp033. 19151095 PMC2672624

[ref46] Kumar S.; Stecher G.; Li M.; Knyaz C.; Tamura K. MEGA X: Molecular evolutionary genetics analysis across computing platforms. Mol. Biol. Evol. 2018, 35, 1547–1549. 10.1093/molbev/msy096. 29722887 PMC5967553

[ref47] Jones D. T.; Taylor W. R.; Thornton J. M. The rapid generation of mutation data matrices from protein sequences. Comput. Appl. Biosci. 1992, 8, 275–282. 10.1093/bioinformatics/8.3.275. 1633570

[ref48] Agirre J.; Moroz O.; Meier S.; Brask J.; Munch A.; Hoff T.; Andersen C.; Wilson K. S.; Davies G. J. The structure of the AliC GH13 α-amylase from *Alicyclobacillus* sp. reveals the accommodation of starch branching points in the α-amylase family. Acta Crystallogr. D. Struct. Biol. 2019, 75, 1–7. 10.1107/S2059798318014900. 30644839 PMC6333287

[ref49] Offen W. A.; Viksoe-Nielsen A.; Borchert T. V.; Wilson K. S.; Davies G. J. Three-dimensional structure of a variant ‘Termamyl-like’ *Geobacillus stearothermophilus* α-amylase at 1.9 Å resolution. Acta Crystallogr. F. Struct. Biol. Commun. 2015, 71, 66–70. 10.1107/S2053230X14026508. 25615972 PMC4304751

[ref50] Yang W.; Su L.; Wang L.; Wu J.; Chen S. α-Glucanotransferase from the glycoside hydrolase family synthesizes α(1–6)-linked products from starch: Features and synthesis pathways of the products. Trends Food Sci. Technol. 2022, 128, 160–172. 10.1016/j.tifs.2022.08.001.

[ref51] Li X.; Wang Y.; Mu S.; Ji X.; Zeng C.; Yang D.; Dai L.; Duan C.; Li D. Structure, retrogradation and digestibility of waxy corn starch modified by a GtfC enzyme from *Geobacillus* sp. 12AMOR1. Food Bioscience 2022, 46, 101527 10.1016/j.fbio.2021.101527.

[ref52] Yang W.; Sheng L.; Su L.; Chen S.; Wu J. Directed mutation of two key amino acid residues alters the product structure of the new 4,6-α-glucanotransferase from *Bacillus sporothermodurans*. J. Agric. Food Chem. 2021, 69, 14680–14688. 10.1021/acs.jafc.1c05263. 34845909

[ref53] Kelly G.; Prasannan S.; Daniell S.; Fleming K.; Frankel G.; Dougan G.; Connerton I.; Matthews S. Structure of the cell-adhesion fragment of intimin from enteropathogenic *Escherichia coli*. Nat. Struct. Biol. 1999, 6, 313–318. 10.1038/7545. 10201396

[ref54] Kurochkina N.; Guha U. SH3 domains: modules of protein-protein interactions. Biophys. Rev. 2013, 5, 29–39. 10.1007/s12551-012-0081-z. 28510178 PMC5418429

[ref55] Vujičić-Žagar A.; Pijning T.; Kralj S.; Lopez C. A.; Eeuwema W.; Dijkhuizen L.; Dijkstra B. W. Crystal structure of a 117 kDa glucansucrase fragment provides insight into evolution and product specificity of GH70 enzymes. Proc. Natl. Acad. Sci. U.S.A. 2010, 107, 21406–21411. 10.1073/pnas.1007531107. 21118988 PMC3003066

[ref56] Arnal G.; Cockburn D. W.; Brumer H.; Koropatkin N. M. Structural basis for the flexible recognition of α-glucan substrates by *Bacteroides thetaiotaomicron* SusG. Protein Sci. 2018, 27, 1093–1101. 10.1002/pro.3410. 29603462 PMC5980535

