# Supplementary material for: Crystal Structure of 4,6-α-Glucanotransferase GtfC-ΔC from Thermophilic Geobacillus 12AMOR1: Starch Transglycosylation in Non-Permuted GH70 Enzymes
Source: J Agric Food Chem. 2022 Nov 28;70(48):15283–95. doi: 10.1021/acs.jafc.2c06394 (PMC9732880; doi:10.1021/acs.jafc.2c06394)
Supplement: Supplementary file 1 — jf2c06394_si_001.pdf [file jf2c06394_si_001.pdf]

SUPPORTING INFORMATION

Manuscript title:

Crystal structure of 4,6-α-glucanotransferase GtfC-ΔC from thermophilic *Geobacillus* 12AMOR1: starch transglycosylation in non-permuted GH70 enzymes

Authors:

Tjaard Pijning[a,*], Evelien M. te Poele[b,c], Tijn C. de Leeuw[c], Albert Guskov[a] and Lubbert Dijkhuizen[b,c]

[a]Biomolecular X-ray Crystallography, Groningen Biomolecular Sciences and Biotechnology Institute (GBB), University of Groningen, Nijenborgh 7, 9747 AG Groningen, The Netherlands

[b]Microbial Physiology, Groningen Biomolecular Sciences and Biotechnology Institute (GBB), University of Groningen, Nijenborgh 7, 9747 AG Groningen, The Netherlands

[c]CarbExplore Research B.V., Zernikelaan 8, 9747 AA Groningen, The Netherlands

*Corresponding author: Tjaard Pijning, Biomolecular X-ray Crystallography, Groningen Biomolecular Sciences and Biotechnology Institute (GBB), University of Groningen, Nijenborgh 7, 9747 AG Groningen, The Netherlands, tel.: +31503634385, fax: +31503634800. Email: t.pijning@rug.nl

Figure S1.

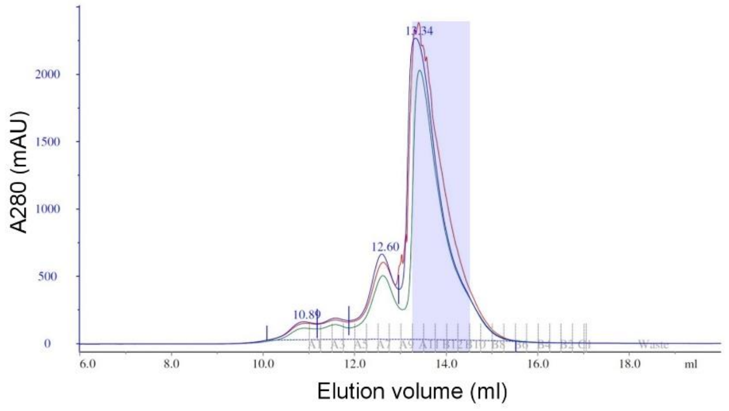

Elution profile of three size exclusion chromatography (SEC) runs with GbGtfC-ΔC on a Superdex 200 10/300 Increase column. The main peak eluting at 13.3 ml corresponds to an apparent MW of 91 kDa; the shaded area was pooled and concentrated to obtain the protein solution used for crystallization experiments.

Figure S2.

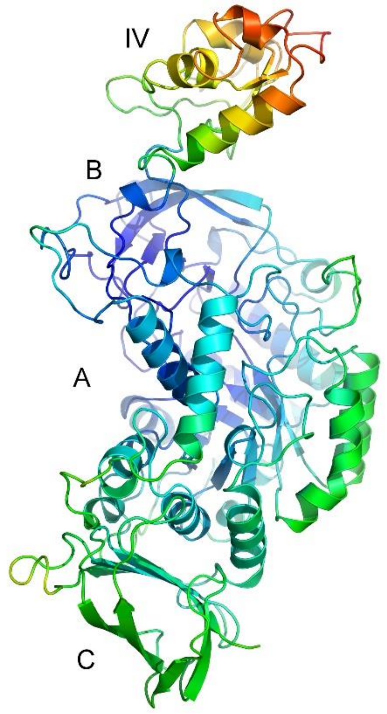

Crystal structure of GbGtfC-ΔC colored by crystallographic B-factors of the Cα atoms (blue = low, red = high), showing the relatively high B-factors of domains IV and C, indicating possible flexibility.

Figure S3.

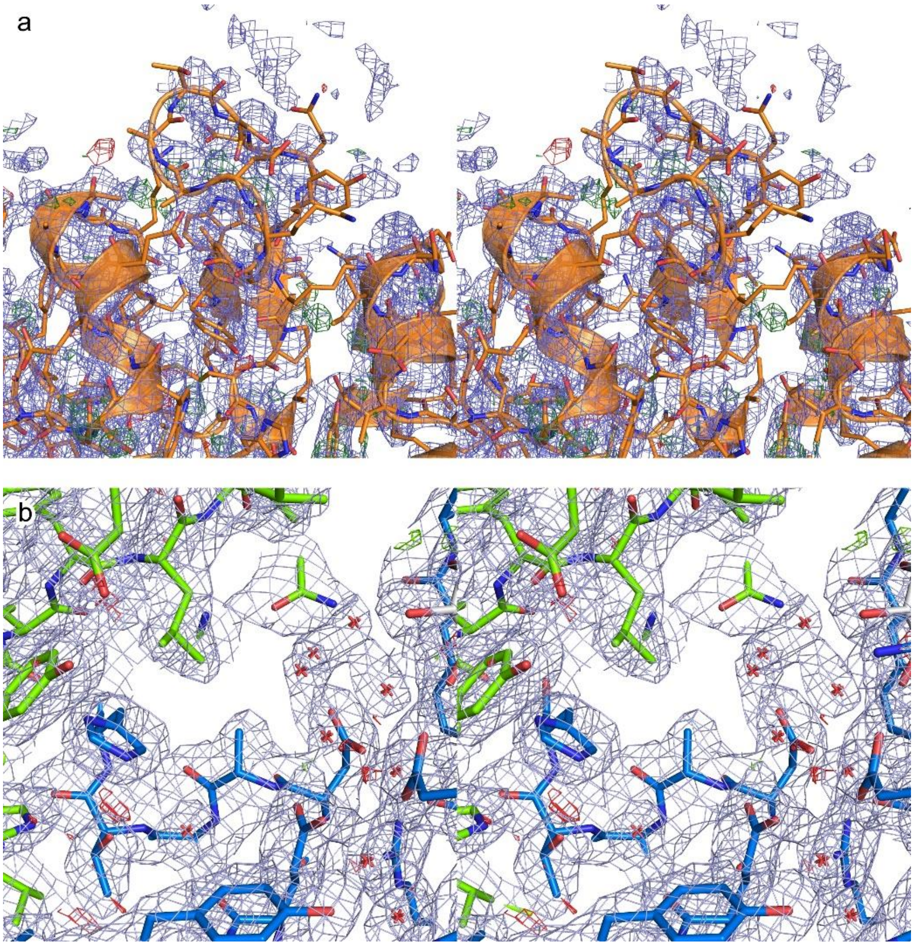

Stereo figures of the GbGtfC crystal structure and electron density; the 2Fo-Fc map is contoured at 1.0 σ (blue), and the Fo-Fc map at +3.0 σ (green) and -3.0 σ (red). (a) Part of domain IV. (b) Part of the active site.

Figure S4

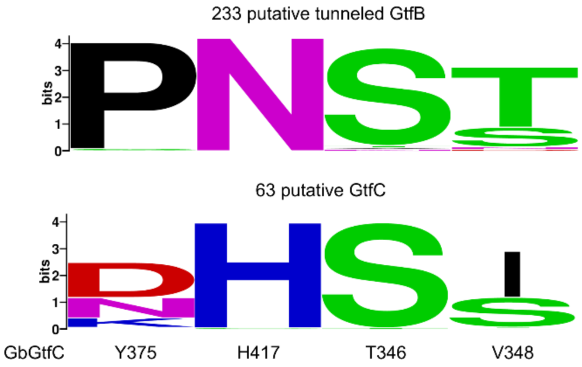

Sequence logos of four non-conserved residues near acceptor subsites +1 and +2 in 63 putative GbGtfC sequences, compared with the corresponding positions in 233 putative GtfB sequences that likely feature a tunneled binding groove with long loops A1 and B. Among the GtfC sequences, GbGtfC is unique at three of the four positions (Y375, T346 and V348, indicated below the sequence logo of GtfCs) while H417 is conserved among GtfC sequences but different from GtfB sequences. The figure was generated with WebLogo.[57]

Molecular docking

Methods

Substrates for docking were setup with SWEET2,[41] modeling variations of (mixed) (isomalto-)maltooligosaccharides with a degree of polymerization (DP) of 1-6; ligands were then prepared using AutoDock Tools (version 1.5.6).[42] Crystal structures of Gb GtfC-ΔC and Lr121 GtfB were also prepared for docking in AutoDock Tools by removing all waters and preparing boxes of size 40 x 40 x 40 for smaller ligands and 20 x 20 x 20 for larger ligands, centered on subsite -1. A covalent glycosyl-enzyme intermediate was prepared in PyMOL by attaching a cyclohexyl group to the catalytic aspartate (D413), and converting it to a glucosyl moiety by attaching -OH and -CH_2OH groups and inserting the ring oxygen. Subsequently, the torsion angles of the D413 side chain were adjusted in order to optimize the CH-π interactions of the attached glucosyl moiety with Y95 below subsite -1, as well as to optimize the glucosyl O6 interaction with D413. Docking was performed with Vina-Carb[43] with an exhaustiveness parameter of 20, and 100 returned results (individual poses). For donor substrate docking, the results (using free enzyme) were visually inspected in PyMOL for D413-OD1 distance to the C1 carbon for glucose and short oligomers, after which longer oligomers were grouped by similarity to these conformations. For acceptor substrate docking (using the covalent

intermediate) the results were accepted if the distance between C1 (of the covalent intermediate) and the nearest substrate oxygen was <3.65 Å. The results were then ordered first by the number of interactions between the substrate and the acid-base (E446) and transition state-stabilizing residue (D517) with a distance <3.05 Å, and second by the Vina-Carb score (based on binding energy). The thus obtained best scoring poses were used for Figure S5. Construction of the glucosyl intermediate, visual inspection of donor substrates and acceptor filtering methods relied on the findings of Jaña *et al*.[58] for *Streptococcus mutans* glucansucrase GTF-SI using quantum mechanics/molecular mechanics (QM/MM) methods, noting that hydrogen bonding to catalytic residues provides significant transition-state stabilizing effects. All docking results were visually inspected in PyMOL and grouped by visual similarity, into conformations like or unlike a pre-transition state, within the above-described distance bounds.

General observations

The docking results support the proposed contribution of non-conserved residues near subsites +1 and +2, although we cannot exclude that other residues play a role in determining reaction specificity. The T346/V348 pair of GbGtfC is somewhat more hydrophobic and closer to docked substrates than the corresponding S918/T920 pair of Lr121 GtfB. Together with the H417 being closer than P968, subsite +1 is more constrained in GbGtfC (Figure S5a), and this likely affects freedom for substrates to adopt favorable conformations for cleavage and/or transglycosylation. For example, the destabilizing interactions of GbGtfC observed in subsites +1 and +2, most significantly by V348, likely contribute to its low propensity for α-1,4-transglycosylation (Table S3). Similarly, destabilizing interactions in Lr121 GtfB by residues T920 (subsite +1) and N1019 (subsite +2) may explain the low reactivity of Lr121 GtfB in α-1,6-donor reactions.

Surprisingly, the docking experiments showed that the donor half-reaction in GbGtfC and Lr121 GtfB is not restricted entirely to α-1,4 cleavage, and actually often favors α-1,6 cleavage. For example, docking of the trisaccharide isopanose in free GbGtfC resulted in two sets of conformations positioning either its α-1,4 or its α-1,6 linkage in the catalytic subsites (-1/+1). These two sets show comparable binding energies (ΔG <0.1 kcal/mol) and substrate geometries (RMSD = 0.011 Å for torsions and 0.60 Å for atom positions), suggesting that they may easily interconvert, by sliding through the binding groove. In the first set (Figure S5b, left panel), the conserved motif III residue Y448 provides aromatic stacking interactions to the reducing (R) end glucosyl moiety; however, the maltosyl moiety shows a geometry unfavorable for cleavage.

In contrast, in the second set, the isomaltosyl moiety occupies the catalytic subsites (-1/+1) in a pre-transition state like conformation, enabling GbGtfC to cleave isopanose at the α-1,6 linkage (Figure S5b, right panel). In Lr121 GtfB, isopanose also can take up positions the place either the α-1,6- or α-1,4-linkage for cleavage (Figure S5c, left and right panel, resp.), but the binding energies and conformation of these positions differ significantly more (ΔG = 0.6 kcal/mol; RMSD = 0.41 Å for torsions and 1.66 Å for atom positions).

Also regarding acceptor reactions, we found that - although it depends on the substrate - transglycosylation is not restricted to one linkage type: not only α-1,6 linkage formation is possible, but also α-1,4. For example, in GbGtfC, a feasible pre-transition state conformation for maltose was observed that positions its 4-OH for attack on the covalent intermediate (Figure 5a, left panel). Notably, the subsite +1 glucosyl moiety has a conformation not very different from that observed in a maltotetraose donor substrate, suggesting that the α-1,4-specific donor and acceptor reactions are more or less reversible: the (intermediate) products of α-1,4 transglycosylation events react back as they easily become α-1,4-specific donor substrates. In contrast, this is not the case for α-1,6-specific half-reactions: here, the conformations of the +1 glucosyl moiety in donor and acceptor substrates clearly differ, requiring a ~180° rotation about the C5-C6 bond (Figures 5a, right panel). Similar observations were made in Lr121 GtfB (not shown). Thus, while the α-1,4 specific half-reactions are reversible and 'cancel eachother out', this is not the case for α-1,6 specific half-reactions.

Differences in donor reaction specificity

Docking results with the trisaccharides panose and isopanose revealed differences in donor reaction specificity between GbGtfC and Lr121 GtfB. As stated above, GbGtfC preferably cleaves the α-1,6 linkage of isopanose; the conformationally (and energetically) similar poses shown in Figure S5b suggest that substrates containing a non-reducing (NR) end isopanosyl moiety may easily 'slide' through the binding groove to place its α-1,6 linkage at the cleavage site. In Lr121 GtfB, the isopanose binding conformations shown in Figure S5c are quite different and require a significant change of torsion angles in the α-1,6 glycosidic linkage, suggesting that this enzyme is less efficient in removing α-1,6-linked maltosyl from the NR end. For panose, no cleavable conformations were found for either the α-1,4 or the α-1,6 linkage with both enzymes. Notably however, attaching an extra 1,4-linked glucosyl unit at the non-reducing end (yielding 6-*O*-α-D-maltosyl-maltose) changes the orientation of the subsite +1 sugar unit and 'repositions' the substrate into an α-1,6-cleavable conformation (Figure S5d).

Together, these results show the preference of GbGtfC to remove α-1,6-linked maltosyl units from NR ends.

Differences in acceptor reaction specificity

As discussed above, transglycosylation can take place with both α-1,4- and α-1,6-specificity. Docking experiments involving glucose, maltose or maltotriose in acceptor reaction scenarios showed such dual specificity (e.g., see Figure 5a for α-1,4- and α-1,6-acceptor reactions of maltose in GbGtfC). On the other hand, maltooligosaccharide acceptor substrates of DP ≥4 showed a clear preference for conformations prone to α-1,6 transglycosylation, while α-1,4 specific conformations seemed incompatible, e.g., due to the reducing end 1-OH group pointing 'into' the protein without room for extending the chain. This preference for α-1,6 transglycosylation was also observed for isomaltose, isomaltotriose, panose, isopanose and isomaltotetraose.

Figure S5.

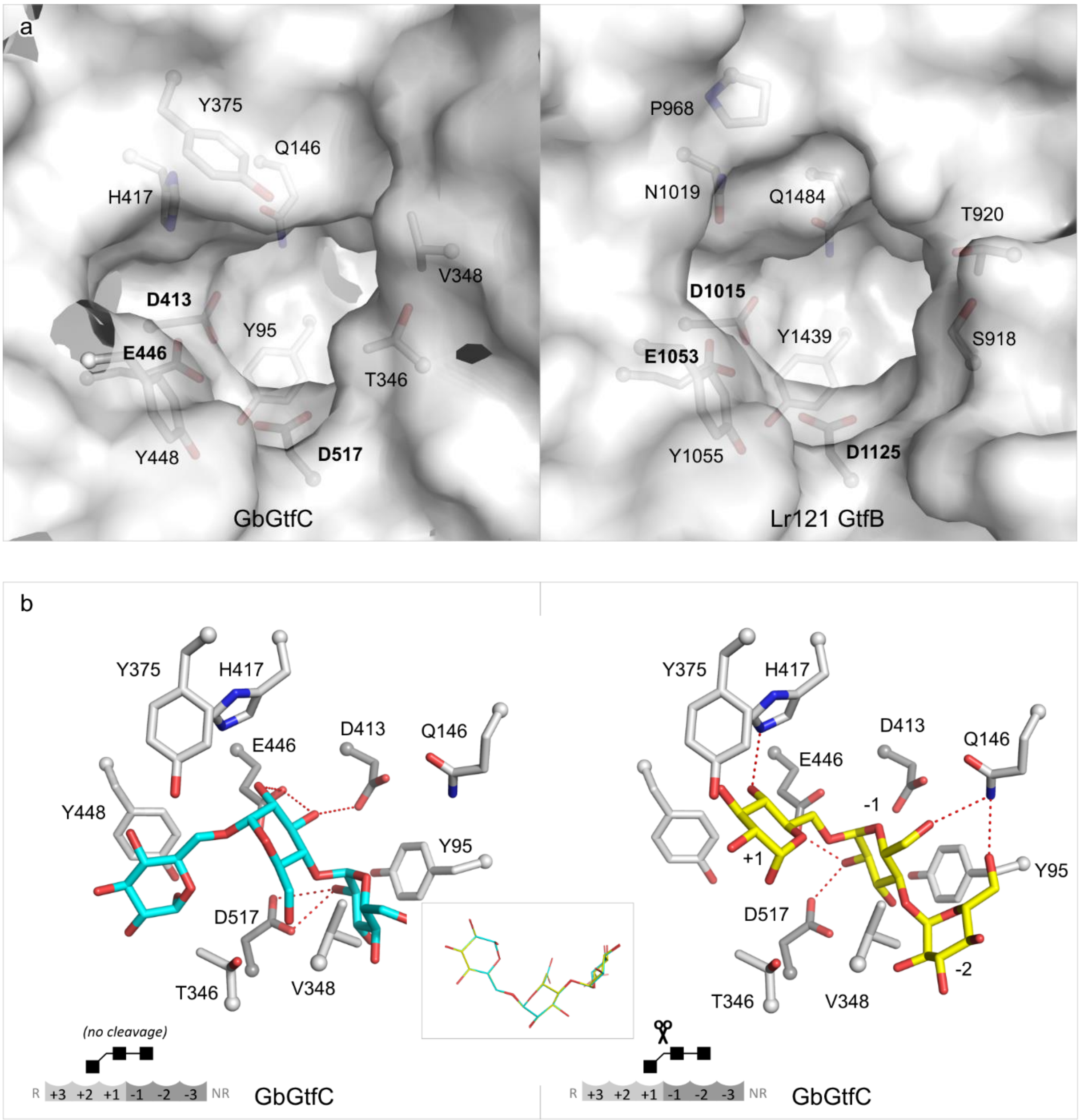
a
Y375
Q146
H417
V348
D413
Y95
E446
T346
Y448
D517
GbGtfC
P968
N1019
Q1484
T920
D1015
Y1439
S918
E1053
Y1055
D1125
Lr121 GtfB
b
Y375
H417
D413
Q146
E446
Y448
Y95
D517
T346
V348
(no cleavage)
R +3 +2 +1 -1 -2 -3 NR
GbGtfC
Y375
H417
D413
Q146
E446
-1
+1
Y95
D517
-2
T346
V348
R +3 +2 +1 -1 -2 -3 NR
GbGtfC

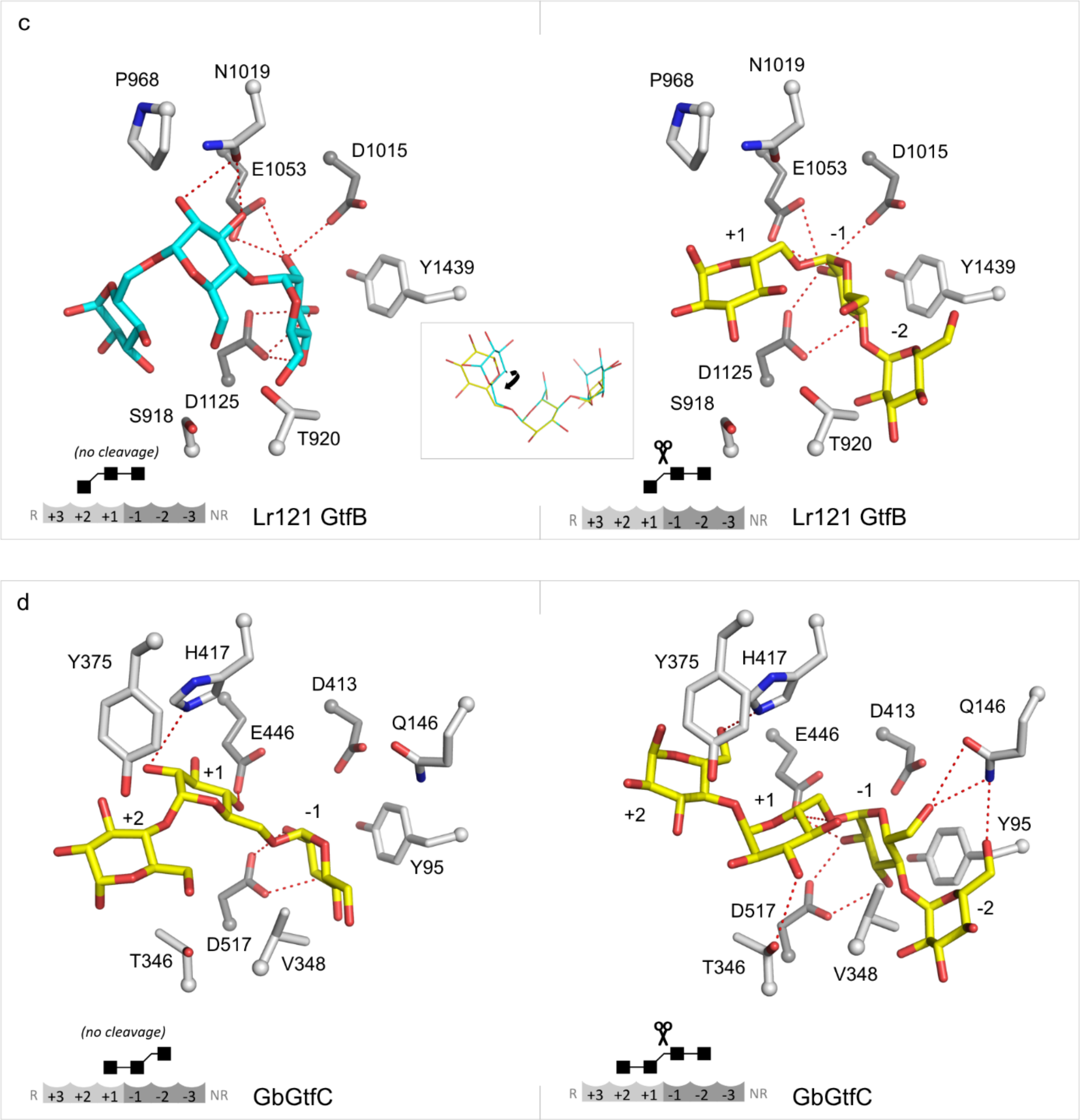

Selected docking results for donor- and acceptor reactions in GbGtfC and Lr121 GtfB; a schematic representation is given with each panel. R and NR designate the reducing resp. non-reducing end of the ligands. Putative hydrogen bonds were assessed by inspection of the models in COOT[34] and are indicated as red dashed lines. (a) Comparison of the accessibility of the active sites of GbGtfC (left) and Lr121 GtfB (right), showing the wider subsite +1/+2 of GbGtfC. (b) α-1,6 Donor specificity in GbGtfC: isopanose assumes a 'half-subsite' pose incompatible for α-1,4 cleavage (left), but its α-1,6-linkage can be cleaved (right). The inset shows the almost identical conformation of both poses. (c) A similar phenomenon is observed in Lr121 GtfB, but here the respective conformations are significantly different (inset). (d) GbGtfC prefers to cleave maltosyl units from the non-reducing end: while panose α-1,6

cleavage is unfavorable (left), attaching an extra sugar unit at the non-reducing end (6'O-α-maltosyl-maltose) orients the +1 sugar unit in such a way that cleavage of the α-1,6 linkage is promoted (right).

Figure S6

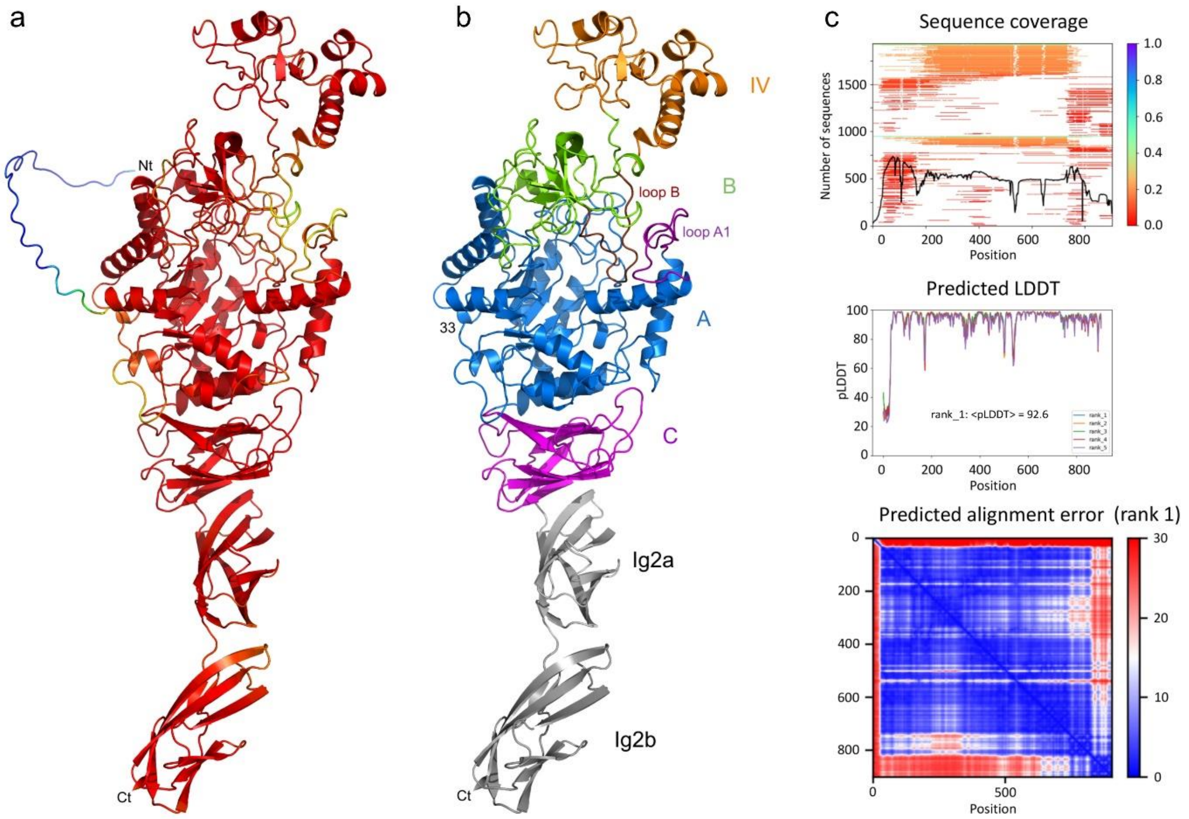

AlphaFold model of GbGtfC. (a) Structure of the highest ranked model (rank 1) colored by pLDDT score (red=high, blue=low); the N-terminal ≈ 30 residues are not modeled confidently. (b) Residues 33-903 of the AlphaFold model colored by domain as in Figure 1 with the two Ig2-type domains in grey; the N-terminal residues 1-32 were omitted from this representation. (c) Per-residue plots for sequence coverage, pLDDT (confidence) and alignment error; the latter suggests that the relative position of domain Ig2a (residues 738-823) and especially domain Ig2b (residues 824-903) is modeled with less confidence, indicated by the red color in this plot.

Figure S7

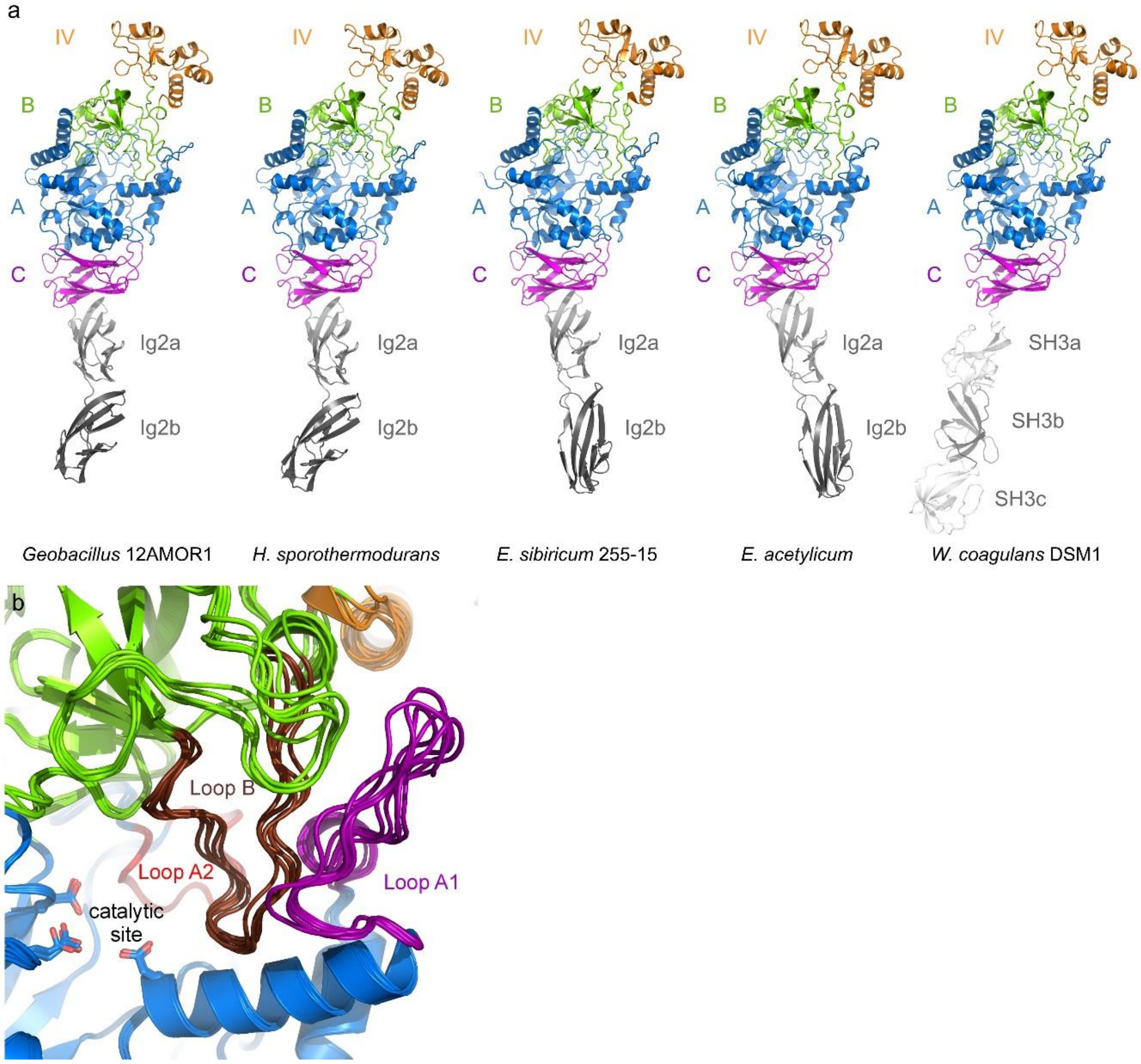

Comparison of AlphaFold models of GbGtfC and four other GtfC-type GTs. (a) Overall structures; N-terminal residues with pLDDT scores <60 are not shown. (b) Superposition of the active sites, with the catalytic residues shown as sticks, and loops A1, A2 and B indicated in purple, brown and red, respectively.

Table S1.

AlphaFold models of selected GtfC-type GTs with their relative sequence identity (seq. id.); for the calculation of the average per-residue confidence score (pLDDT), N-terminal residues with a pLDDT <60 were omitted. Root mean square deviation (RMSD) values were calculated from superposition with GbGtfC using Cα atoms.

**Database entry**	**Bacterial species**	**Seq. id. (%)**	**pLDDT**	**RMSD (Å)**
AKM18207.1	*Geobacillus* 12AMOR1	100.0	94.8	-
WP_066235061.1	*Heyndrickxia sporothermodurans*	76.3	94.9	0.61
WP_012371512.1	*Exiguobacterium sibiricum* 255-15	56.3	94.6	0.66
WP_029342707.1	*Exiguobacterium acetylicum*	56.5	94.3	0.72
WP_234969614.1	*Weizmannia coagulans* DSM-1	72.6	94.8	0.54

Table S2.

List of sequences used for the alignment of 121 GH70/GH13 enzymes using the GbGtfC sequence as reference (sequence no. 1): sequences 2-102 found from a BLAST search, containing (putative) GtfC- and GtfD-type α-glucanotransferases. Other sequences were added: α-amylases from GH13_5 (sequences 103-110), GtfB-type α-glucanotransferases (sequences 111-116) and glucansucrases / branching sucrases (sequences 116-121). Black dots indicate the sequences for which an AlphaFold model was calculated (see also Table S1). For the GH70 α-GTs, the lengths for loops A1 and B of GtfB are indicated (for GtfB enzymes the lengths were taken from Pijning *et al.*[26]

**No.**	**Database entry**	**Bacterial species**	**(Sub)family**	**Enzyme name**	**Sequence length**	**Sequence id.** (%)	**Length loop A1**	**Length loop B**
1	AKM18207.1	*Geobacillus sp.* 12AMOR1 ●	GH70 GtfC	GbGtfC	903	100.0	21	15
2	WP_066235061.1	*Heyndrickxia sporothermodurans* ●	GH70 GtfC		902	76.30	21	15
3	WP_202757886.1	*Heyndrickxia sporothermodurans*	GH70 GtfC		902	76.19	21	15
4	WP_035322188.1	*Peribacillus kribbensis*	GH70 GtfC		904	65.45	21	15
5	WP_235962329.1	*Weizmannia coagulans*	GH70 GtfC		888	72.55	21	15
6	*NMH84024.1*	*Weizmannia coagulans*	GH70 GtfC		877	73.55	21	15
7	WP_237340457.1	*Weizmannia coagulans*	GH70 GtfC		889	72.94	21	15
8	WP_235601145.1	*Weizmannia coagulans*	GH70 GtfC		889	72.94	21	15
9	AVD57793.1	*Weizmannia coagulans*	GH70 GtfC		877	74.48	21	15
10	WP_230951688.1	*Weizmannia coagulans*	GH70 GtfC		888	73.17	21	15
11	KWZ85978.1	*Weizmannia coagulans*	GH70 GtfC		889	73.23	21	15
12	MBF8417483.1	*Weizmannia coagulans*	GH70 GtfC		876	73.24	21	15

13	WP_233896645.1	*Weizmannia coagulans*	GH70 GtfC		889	73.85	21	15
14	WP_237342445.1	*Weizmannia coagulans*	GH70 GtfC		889	72.81	21	15
15	WP_237348871.1	*Weizmannia coagulans*	GH70 GtfC		888	72.55	21	15
16	WP_017553304.1	*Weizmannia coagulans*	GH70 GtfC		876	73.01	21	15
17	WP_235920097.1	*Weizmannia coagulans*	GH70 GtfC		888	72.76	21	15
18	WP_029141257.1	*Weizmannia coagulans*	GH70 GtfC		742	73.62	21	15
19	NCG69168.1	*Weizmannia coagulans*	GH70 GtfC		876	73.48	21	15
20	WP_244490584.1	*Weizmannia coagulans*	GH70 GtfC		748	71.87	21	15
21	QJE33912.1	*Weizmannia coagulans*	GH70 GtfC		953	73.14	21	15
22	WP_235601561.1	*Weizmannia coagulans*	GH70 GtfC		763	72.81	21	15
23	AEH52441.1	*Weizmannia coagulans* 2-6	GH70 GtfC		954	72.68	21	15
24	WP_234969614.1	*Weizmannia coagulans* DSM-1 ●	GH70 GtfC		965	72.55	21	15
25	KYC62708.1	*Weizmannia coagulans*	GH70 GtfC		954	73.83	21	15
26	WP_235601886.1	*Weizmannia coagulans*	GH70 GtfC		966	73.21	21	15
27	WP_118500555.1	*Weizmannia coagulans*	GH70 GtfC		875	74.03	21	15
28	WP_118499016.1	*Weizmannia coagulans*	GH70 GtfC		875	72.97	21	15
29	WP_214892803.1	*Exiguobacterium* H66	GH70 GtfC		893	57.22	22	15
30	WP_214807082.1	*Exiguobacterium* s102	GH70 GtfC		893	57.22	22	15
31	WP_214721726.1	*Exiguobacterium* s192	GH70 GtfC		893	57.17	22	15
32	WP_028105602.1	*Exiguobacterium undae*	GH70 GtfC		893	57.17	22	15
33	WP_012371512.1	*Exiguobacterium sibiricum* 255-15 ●	GH70 GtfC	EsGtfC	893	56.32	22	15
34	WP_214849840.1	*Exiguobacterium* s138	GH70 GtfC		893	56.61	22	15
35	WP_214846289.1	*Exiguobacterium* S90	GH70 GtfC		893	56.19	22	15
36	WP_214853423.1	*Exiguobacterium* s166	GH70 GtfC		893	56.39	22	15
37	WP_026830256.1	*Exiguobacterium antarcticum*	GH70 GtfC		893	56.84	22	15
38	WP_014971370.1	*Exiguobacterium antarcticum*	GH70 GtfC		893	56.95	22	15
39	WP_071499881.1	*Exiguobacterium* KRL4	GH70 GtfC		893	56.84	22	15
40	WP_159173340.1	*Exiguobacterium* 9Y	GH70 GtfC		893	57.00	22	15
41	WP_209548227.1	*Exiguobacterium* unclassified	GH70 GtfC		893	57.06	22	15
42	WP_035410561.1	*Exiguobacterium* RIT341	GH70 GtfC		892	56.59	22	15
43	WP_188004779.1	*Exiguobacterium helios*	GH70 GtfC		893	56.10	22	15
44	WP_149427900.1	*Exiguobacterium acetylicum*	GH70 GtfC		892	56.94	22	15
45	WP_214771614.1	*Exiguobacterium* unclassified	GH70 GtfC		892	57.24	22	15
46	WP_064299263.1	*Exiguobacterium* KKBO11	GH70 GtfC		892	56.71	22	15
47	WP_214805596.1	*Exiguobacterium* s46	GH70 GtfC		892	56.71	22	15
48	WP_214724116.1	*Exiguobacterium* s143	GH70 GtfC		892	56.71	22	15
49	WP_214852153.1	*Exiguobacterium* s130	GH70 GtfC		892	56.59	22	15
50	WP_215147047.1	*Exiguobacterium* s91	GH70 GtfC		892	56.71	22	15
51	WP_214858400.1	*Exiguobacterium* s191	GH70 GtfC		892	56.71	22	15
52	WP_026827371.1	*Exiguobacterium sibiricum*	GH70 GtfC		893	56.61	22	15
53	WP_050678213.1	*Exiguobacterium acetylicum*	GH70 GtfC		892	56.71	22	15
54	WP_047390368.1	*Exiguobacterium*	GH70 GtfC		892	56.38	22	15
55	WP_069940018.1	*Exiguobacterium* U13-1	GH70 GtfC		892	56.49	22	15
56	WP_029342707.1	*Exiguobacterium acetylicum* ●	GH70 GtfC		892	56.49	22	15
57	WP_247856602.1	*Exiguobacterium* 17-1	GH70 GtfC		893	56.39	22	15
58	WP_214807949.1	*Exiguobacterium* s144	GH70 GtfC		892	56.82	22	15
59	WP_058704108.1	*Exiguobacterium indicum*	GH70 GtfC		892	56.49	22	15
60	WP_223040902.1	*Exiguobacterium acetylicum*	GH70 GtfC		892	56.60	22	15
61	HCV52719.1	*Exiguobacterium*	GH70 GtfC		837	57.21	22	15
62	WP_239984758.1	*Sporolactobacillus pectinivorans*	GH70 GtfC		1022	52.91	21	15
63	HAB33825.1	*Exiguobacterium*	GH70 GtfC		711	55.18	22	15
64	WP_214713558.1	*Bacillus* unclassified	GH70 GtfD		783	49.51	13	15
65	WP_214732158.1	*Bacillus* ISL-46	GH70 GtfD		783	49.51	13	15
66	WP_052702730.1	*Paenibacillus beijingensis*	GH70 GtfD	PbGtfD	776	48.28	13	15
67	WP_224722266.1	*Paenibacillus* N4	GH70 GtfD		777	48.34	13	15
68	WP_142092843.1	*Propioniferax innocua*	GH70 GtfD		1011	49.65	21	15
69	WP_223067679.1	*Paenibacillus caui*	GH70 GtfD		777	49.93	13	15
70	WP_175890816.1	*Burkholderia*	GH70 GtfD		721	44.95	13	15
71	WP_175908991.1	*Burkholderia* BCC1640	GH70 GtfD		721	44.82	13	15
72	WP_175712399.1	*Burkholderia ambifaria*	GH70 GtfD		721	45.03	13	15
73	WP_205786286.1	*Burkholderia* Ac-20344	GH70 GtfD		721	46.04	13	15
74	WP_174393660.1	*Burkholderia cepacia*	GH70 GtfD		721	44.77	13	15

75	WP_119338563.1	*Burkholderia*	GH70 GtfD		721	45.21	13	15
76	WP_091925906.1	*Burkholderia*	GH70 GtfD		721	45.21	13	15
77	SEU46829.1	*Burkholderia cepacia*	GH70 GtfD		686	45.93	13	15
78	WP_175911446.1	*Burkholderia* unclassified	GH70 GtfD		727	40.42	13	15
79	WP_158592123.1	*Pseudomonas cavernicola*	GH70 GtfD		780	44.75	14	15
80	RJG09097.1	*Pseudomonas cavernicola*	GH70 GtfD		797	44.75	14	15
81	WP_243862281.1	*Frigoribacterium faeni*	GH70 GtfD		941	41.06	11	15
82	NIJ05635.1	*Frigoribacterium faeni*	GH70 GtfD		923	41.63	11	15
83	WP_049623289.1	*Frateuria defendens*	GH70 GtfD		790	42.52	13	15
84	NVK44519.1	*Oceanospirillaceae bacterium*	GH70 GtfD		775	42.52	14	15
85	WP_167520052.1	*Azotobacter salinestris*	GH70 GtfD		742	41.93	14	15
86	WP_240931651.1	*Azotobacter chroococcum*	GH70 GtfD		695	41.79	14	15
87	NHN78727.1	*Azotobacter chroococcum*	GH70 GtfD		736	41.79	14	15
88	WP_198318972.1	*Azotobacter chroococcum*	GH70 GtfD		736	41.52	14	15
89	WP_162502917.1	*Azotobacter chroococcum*	GH70 GtfD		736	41.38	14	15
90	WP_169531329.1	*Azotobacter chroococcum*	GH70 GtfD		759	41.52	14	15
91	TKD39461.1	*Azotobacter chroococcum*	GH70 GtfD		780	41.52	14	15
92	TBW10122.1	*Azotobacter chroococcum subsp. isscasi*	GH70 GtfD		780	41.38	14	15
93	TBW10924.1	*Azotobacter chroococcum*	GH70 GtfD		780	41.52	14	15
94	WP_207388900.1	*Azotobacter chroococcum*	GH70 GtfD		759	41.52	14	15
95	WP_158319672.1	*Azotobacter chroococcum*	GH70 GtfD		731	41.52	14	15
96	TBV96991.1	*Azotobacter chroococcum*	GH70 GtfD		782	41.52	14	15
97	WP_198866748.1	*Azotobacter chroococcum*	GH70 GtfD		759	41.24	14	15
98	OHC12359.1	*Pseudomonadales bacterium* GWC1_66_9	GH70 GtfD		731	41.52	14	15
99	WP_165494383.1	*Azotobacter chroococcum*	GH70 GtfD		759	41.52	14	15
100	WP_165496545.1	*Azotobacter chroococcum*	GH70 GtfD		743	41.52	14	15
101	TBW34927.1	*Azotobacter chroococcum*	GH70 GtfD		780	41.52	14	15
102	AJE22990.1	*Azotobacter chroococcum* NCIMB 8003	GH70 GtfD	AcGtfD	780	41.52	14	15
103	A0A3P8MUS3	*Alicyclobacillus*	GH13_5					
104	AAA22191.1	*Bacillus amyloliquefaciens*	GH13_5					
105	AAA22226.1	*Bacillus licheniformis*	GH13_5					
106	AEM05860.1	*Bacillus licheniformis*	GH13_5					
107	AAA22231.1	*Bacillus* 707	GH13_5					
108	Q93I48	*Bacillus* KSM K38	GH13_5					
109	P06279	*Geobacillus stearothermophilus*	GH13_5					
110	AIV43245.1	*Geobacillus stearothermophilus*	GH13_5					
111	ASA47879.1	*Limosilactobacillus reuteri* NCC 2613	GH70 GtfB	Lr2613 GtfB			10	4
112	WP_082229424.1	*Limosilactobacillus fermentum* NCC 2970	GH70 GtfB	Lf2970 GtfB			13	8
113	Q5SBM0	*Limosilactobacillus reuteri* 121	GH70 GtfB	Lr121 GtfB			17	20
114	ASA47903.1	*Streptococcus thermophilus*	GH70 GtfB	St GtfB			13	8
115	KRM39239.1	*Ligilactobacillus aviarius subsp. aviarius* DSM 20655	GH70 GtfB	LaGtfX			17	20
116	KRM39240.1	*Ligilactobacillus aviarius subsp. aviarius* DSM 20655	GH70 GtfB	LaGtfY			17	20
117	Q5SBN3	*Limosilactobacilus reuteri* 180	GH70 GS	Gtf180				
118	Q5SBL9	*Limosilactobacillus reuteri* 121	GH70 GS	GtfA				
119	P13470	*Streptococcus mutans*	GH70 GS	Gtf-SI				
120	A0A2H4A2M1	*Leuconostoc citreum* NRRL B-1299	GH70 BrS	DSR-M				
121	G8XR50	*Leuconostoc mesenteroides* NRRL B-1355	GH70 BrS	ASR				

Table S3.

Correlation between reactivity and subsite +1/+2 glucosyl interactions.

**Enzyme**	**GbGtfC**			**Lr121GtfB**			
Residue and subsite	T346<br>+2	V348<br>+1	H417<br>+1	S918	T920<br>+1	N1019<br>+1	 +2
Acceptor 4'O	- O3/O4	- O	+ O3			+ O2/O3	
Acceptor 6'O					+ O1;<br>- O2/O3	- O3	
Donor α-1,4	O		+ O2		- O6	O3	
Donor α-1,6			+ O2/O3		- O6	+ O4	- O3

Interactions between selected non-conserved residues in GbGtfC and Lr121GtfB and oxygen atoms (O, O2, O3, O4 or O6) of glucosyl moieties in subsites +1 and +2. To arrive at these interactions, the docking results from all 4'O-/6'O-acceptor reactions and α-1,4-/α-1,6 acceptor reactions were clustered. In case there were inconsistent or no interactions, the results are not given. The - and + signs indicate whether the reaction was slow resp. fast, or showed low resp. high similarity to quantum mechanics/molecular mechanics (QM/MM) results from Jaña *et al*.[58] For example, for GbGtfC, 4'O acceptor reactions have a relatively low reactivity; docked acceptor substrates in this scenario in general showed interactions of the acceptor substrate with O (V348, subsite +1) and O3 and O4 with T346 and V348.

References

57. Crooks, G.E.; Hon, G.; Chandonia, J.M.; Brenner, S.E. WebLogo: a sequence logo generator. *Genome Res.* **2004,** *14,* 1188-1190.

58. Jaña, G.A.; Mendoza, F.; Osorio, M.I.; Alderete, J.B.; Fernandes, P.A.; Ramos, M.J.; Jiménez, V.A. A QM/MM approach on the structural and stereoelectronic factors governing glycosylation by GTF-SI from *Streptococcus mutans*. *Org. Biomol. Chem.* **2018,** *16,* 2438-2447.
